# Thioredoxin-interacting protein deficiency protects against severe acute pancreatitis by suppressing apoptosis signal-regulating kinase 1

**DOI:** 10.1038/s41419-022-05355-x

**Published:** 2022-10-31

**Authors:** Yanna Liu, Mengke Li, Chaopeng Mei, Huning Cui, Qiaofang Wang, Dejian Li, Yaodong Song, Mengwei Cui, Qianqian He, Sanyang Chen, Changju Zhu

**Affiliations:** 1 Department of Emergency, The First Affiliated Hospital of Zhengzhou University, No 1 Eastern Jianshe Road, Zhengzhou, 450052 Henan China; 2 Henan Medical Key Laboratory of Emergency and Trauma Research, Zhengzhou, Henan, 450052 China; 3 Henan Emergency and Trauma Medicine Engineering Research Center, Zhengzhou, Henan, 450052 China; 4 Translational Medical Center, The First Affiliated Hospital of Zhengzhou University, Zhengzhou, Henan, 450052 China

**Keywords:** Genetic engineering, Synthetic biology

## Abstract

Acute pancreatitis is a common acute inflammatory abdominal disease. When acute pancreatitis progresses to severe acute pancreatitis (SAP), it can lead to systemic inflammation and even multiple organ failure. Thioredoxin-interacting protein (TXNIP) is an important protein involved in redox reactions of the inflammatory response. However, the specific role of TXNIP in SAP remains unclear. In this study, we investigated the role of thioredoxin interacting protein (TXNIP) in acute pancreatitis when induced by high doses of arginine. We found that pancreatic damage and the inflammatory response associated with acute pancreatitis were largely restrained in TXNIP knock-out mice but were enhanced in mice overexpressing TXNIP. Interestingly, the phosphorylation of p38, JNK, and ASK1 diminished in TXNIP-KO mice with pancreatitis in comparison with wild-type mice. The role of oxidative stress in SAP was explored in two models: TXNIP and AVV-TXNIP. TXNIP knockdown or the inhibition of ASK1 by gs-4997 abrogated the increase in p-p38, p-JNK, and p-ASK1 in AR42J cells incubated with L-Arg. The administration of gs-4997 to mice with pancreatitis largely reduced the upregulation of IL-6, IL-1β, TNF-α, and MCP-1. Systemic inflammatory reactions and injury in the lungs and kidneys were assessed in TXNIP-KO and AVV-TXNIP mice with expected outcomes. In conclusion, TXNIP is a novel mediator of SAP and exerts action by regulating inflammatory responses and oxidative stress *via* the ASK1-dependent activation of the JNK/p38 pathways. Thus, targeting TXNIP may represent a promising approach to protect against SAP.

## Introduction

Acute pancreatitis (AP) refers to a common and acute condition in the abdomen and digestive system. Clinically, most cases of APs involve mild pancreatitis; however, almost 25% of cases have a dangerous onset, severe progression and eventually develop into severe pancreatitis (SAP); these cases are often associated with a systemic inflammatory response syndrome (SIRS) and multiple organ failure (MOF), with a mortality rate of up to 30% [1, 2]. The pathogenesis of SAP has yet to be fully clarified and treatment options are limited. Pancreatic stellate cells (PSCs) are resident cells in the exocrine pancreas and contribute to pancreatic fibrosis and inflammation [3]. Pancreatic stellate cells are usually quiescent, and PSCs secrete neither digestive enzymes nor hormones in contrast to the more abundant acinar cells or islets in the pancreas. Studies have found that activated PSCs contribute to the development of pancreatic fibrosis by depositing collagen fibers in chronic pancreatitis and pancreatic cancer [4, 5]. Studies have demonstrated that PSCs are activated during AP, and are associated with progression from AP to CP [6, 7]. Another study found that the NF-κB signaling pathway in PSC during pancreatitis has a protective immunomodulatory effect [3]. It is well known that Ca^2+^ overload is an important signaling promoting pancreatic necrosis in both acute and chronic pancreatitis, and recent evidence suggests that the physiology of PSCs is also regulated by intracellular Ca^2+^ [8]. Thus, PSCs may signal via Ca^2+^ promotes pancreatic inflammation. Systemic inflammation and oxidative stress are known to be the main pathological processes underlying SAP. It is generally accepted that the pathogenesis of AP is the abnormal activation of a large amount of pancreatic enzymes that cause the pancreas to digest itself, thereby causing a series of inflammatory responses, thus resulting in damage to the pancreas and its surrounding tissues [7]. The local pancreatic activation of transcription factors, recruitment of inflammatory cells, and the production of inflammatory mediators such as TNF-α and IL-1β, further potentiate the local tissue destruction and increase the potential for a life-threatening systemic inflammatory response [9, 10]. Oxidative stress also plays a major role in SAP, as evidenced by diminished reduced glutathione levels, elevated malondialdehyde (MDA) levels, and reduced superoxide dismutase (SOD) activity in the pancreas [11]. In addition to acting as deleterious agents, reactive oxygen species (ROS) are signal-transducing molecules that trigger proinflammatory cytokine production and induce persistent tissue damage in SAP [12]. Therefore, inhibition of the inflammatory response and oxidative stress plays a major role in SAP.

Thioredoxin-interacting protein (TXNIP) is a member of the α-arrestin protein superfamily involved in redox reactions and is associated with the occurrence and development of various diseases [13, 14]. TXNIP binds covalently to thioredoxin and inhibits its ability to remove ROS. In a previous study, Kim et al. found that TXNIP induced redox stress, inflammation, and apoptosis in diabetes and was also associated with the complications of diabetes [15]. Another study showed that melatonin alleviates lung inflammation caused by silica nanoparticles by downregulating TXNIP-related signaling pathways [16]. TXNIP can regulate AP through a variety of signaling pathways, and it was found that diosgenin derivative D can inhibit L-arginine-induced AP by mediating GSDMD in the endoplasmic reticulum through the TXNIP/HIF-1α pathway [17]. Another study found that in lipopolysaccharide (LPS)-induced pancreatic acinar cell (AR42J cell) injury, the expression of TXNIP increased by activating NLRP3-mediated inflammatory pathway and NF-κB signaling pathway [18]. Studies have also found that, baicalein alleviates pyroptosis and inflammation in hyperlipidemic pancreatitis by inhibiting NLRP3/Caspase-1 pathway through the miR-192-5p/TXNIP axis [19]. However, the role of TXNIP in SAP has yet to be evaluated. Therefore, TXNIP is an important molecule connecting various cellular functional pathways in oxidative stress and inflammation [20, 21]. However, the role of TXNIP in SAP has yet to be evaluated.

In this preliminary study, we found that TXNIP expression was significantly upregulated in the pancreas of wild-type (WT) SAP mice and in L-arginine (L-Arg)-treated pancreatic acinar cells, thus suggesting that TXNIP plays a role in SAP. We then used TXNIP-knockout (TXNIP-KO) mice, adeno-associated virus-mediated TXNIP-overexpressing (AAV-TXNIP) mice, and TXNIP knockdown (shTXNIP) mice to verify the role of TXNIP in SAP and identify the underlying mechanisms. Our in vivo and in vitro experiments demonstrated that TXNIP deficiency alleviated SAP by inhibiting the inflammatory response and oxidative stress. Moreover, we found that the protective effect of TXNIP deficiency on SAP depends on the apoptotic signal-regulated kinase 1 (ASK1)-c-Jun N-terminal kinase (JNK)/p38 mitogen-activated protein kinase (p38) pathways. Collectively, these data provide strong evidence for the role of TXNIP in the pathogenesis of AP.

## Materials and methods

### Animals

Male C57BL/6 mice aged 6–8 weeks, weighing 18–22 g, were purchased from the Huaxing Experimental Animal Center (Zhengzhou, China). Mice were housed at 20–25 °C in a 12 h light/dark cycle and fed a standard diet and sterile water. All animals and experimental procedures were approved by the Ethics Committee of the First Affiliated Hospital of Zhengzhou University (Ethical Review Number 2019-KY-140). Male and female heterozygous TXNIP+/− mice (C57BL/6 background) were obtained from Cyagen Biotechnology Co., Ltd. (Jiangsu, China). Mice were acclimatized for 1 week prior to breeding procedures. Genotyping was conducted using genomic DNA extracted from mouse tail tips. TXNIP-KO mice were fed for 2–4 weeks before experiments. To determine the effect of TXNIP on SAP, C57BL/6 mice were injected intraperitoneally with a single dose of AAV9-TXNIP, AAV9-shTXNIP, or control vectors (2 × 10^11^ viral genomes/mouse, diluted to a volume of 200 μl in sterile PBS).

### SAP induction

Before establishment of the SAP model, all mice were fasted for 12 h. L-Arg was dissolved in normal saline and the pH was adjusted to 7 with 10% hydrochloric acid. L-Arg was injected intraperitoneally at a dose of 4 g/kg twice with a 1 h interval. The control group received an intraperitoneal injection of an equal volume of normal saline. After injection, the mice had free access to food and sterile water, and their living conditions remained the same. Seventy-two hours after the induction of AP, the mice were sacrificed. Pancreatic tissue, lung tissue, kidney tissue, and blood were harvested for analysis. For histological analysis, one-third of the pancreatic, lung, and kidney tissues were fixed in 10% neutral-buffered formalin. The remaining tissues were snap-frozen in liquid nitrogen and stored at -80 °C until use. Collected blood samples were centrifuged at 3000 × *g* for 10 min at 4 °C and serum samples were stored at −80 °C until use.

### Enzyme-linked immunosorbent assays

Serum amylase and lipase activity, along with the serum levels of IL-6, IL-1β, TNF-α, and MCP-1 were evaluated by ELISA using commercially available kits in accordance with manufacturer’s instructions.

### Hematoxylin and eosin (H&E) staining and immunohistochemistry (IHC)

H&E staining was performed in paraffin-embedded sections of the pancreas, lungs, and kidneys to observe edema and inflammatory changes in tissues after treatment in the various groups. All tissue sections were assessed under optical light microscopy and pancreatic tissue damage was evaluated in 10 random fields by experienced pathologists blinded to the experimental treatment. Immunohistochemical analysis of TXNIP, cluster of differentiation molecule 11b (CD11b), myeloperoxidase (MPO), lymphocyte antigen 6 complex locus G6D (LY6G), and 4-hydroxynonenal (4-HNE) was performed in paraffin-embedded sections of pancreatic tissue, in accordance with a previously published protocol [22].

### Oxidative stress assay

Pancreatic tissue was taken to prepare pancreatic tissue homogenate and the supernatant was extracted. Then, we used glutathione (GSH), hydrogen peroxide (H_2_O_2_), superoxide dismutase (SOD) and malondialdehyde (MDA) ELISA kits to determine the concentration of BCA protein in accordance with the manufacturer’s instructions.

### Cell culture and treatments

AR42J cells are a rat pancreatic cell line with similarities to acinar cells, AR42J cells have been shown to be a suitable in vitro model for the induction of AP. AR42J cells was cultured in high-glucose F-12K medium containing fetal bovine serum and maintained at 37 °C in a humid environment with 5% CO_2_. Experiments were performed when the cells reached 80–90% confluency. The AR42J cells were treated using L-Arg (10 mg/ml) for 12 h. Then, the cells were collected for analysis. The AR42J cells were transfected with a specific TXNIP siRNA (50 nM) using lipofectamine 3000 transfection reagent in accordance with the manufacturer’s instructions to knockdown TXNIP. Twenty-four hours after transfection, the cells were incubated with L-Arg (10 mg/ml) for analysis. All experiments were repeated in triplicate, at least.

### ROS measurement

We used a ROS Assay Kit to detect ROS in L-Arg-treated AR42J cells. A fluorescence probe (2ʹ,7ʹ-Dichlorodihydrofluorescein diacetate (DCFH-DA)) was diluted in serum-free medium (1:1000) to achieve a final concentration of 10 µmol/l. Cells were then collected and suspended in diluted DCFH-DA at 1 × 10^6^–2 × 10^7^/ml and incubated at 37 °C for 20 min. The mixture was inverted every 3–5 min to bring the probe and cells into full contact. At the end of incubation, cells were washed three times with serum-free medium to remove the DCFH-DA that had not entered the cells. Images were then acquired under a fluorescence microscope (Olympus DX51, Tokyo, Japan).

### Protein extraction and western blotting

Total protein was extracted from pancreatic tissue and AR42J cells by RIPA lysis buffer containing protease and phosphatase inhibitors. the concentrations of total protein samples were then determined with a BCA kit. Equal amounts of protein were separated by sodium dodecyl sulfate-polyacrylamide gel electrophoresis (SDS-PAGE) and then transferred to a polyvinylidene fluoride (PVDF) membrane. The PVDF membrane was blocked with fast blocking solution for 15 min. The membrane was washed three times with Tris-buffered saline with 0.1% Tween 20 (TBST) for 10 min per wash. Then, the membrane was incubated overnight on a shaker at 4 °C with anti-GAPDH (1:1000), -p65 (1:1000), -p-p65 (1:1000), -HO-1 (1:1000), -NQO-1 (1:1000), -p38 (1:1000), -p-p38 (1:500), -JNK (1:1000), -p-JNK (1:500), -ASK1 (1:500), -p-ASK1 (1:500) -ACSL4 (1:1000), or -GPX4 (1:500) antibodies. The membrane was washed three times with TBST for 10 min each wash and then incubated with an HRP-conjugated rabbit or mouse secondary antibody (1:5000) on a shaker for 1 h at room temperature. The membrane was washed again three times with TBST for 10 min each wash. Immune complexes were detected by enhanced chemiluminescence. Images were acquired using the Odyssey Imaging System (LI-COR, Linkon, NE, USA). Protein abundance was assessed in at least three biological replicates. Bands were quantified using Image J software (NIH, Bethesda, MD, USA) with GAPDH as the control.

### Statistical analysis

All data are presented as the mean ± standard deviation (SD). Statistical comparisons were performed using two-tailed Student’s t-tests between two groups or one-way analysis of variance (ANOVA) between three groups. SPSS (version 21.0, IBM, Armonk, NY, USA) was used for these analyses and *p < 0.05, **p < 0.01, ***p < 0.001 was considered statistically significant.

## Results

### TXNIP expression was upregulated after the induction of SAP in mice

**Fig. 1:**
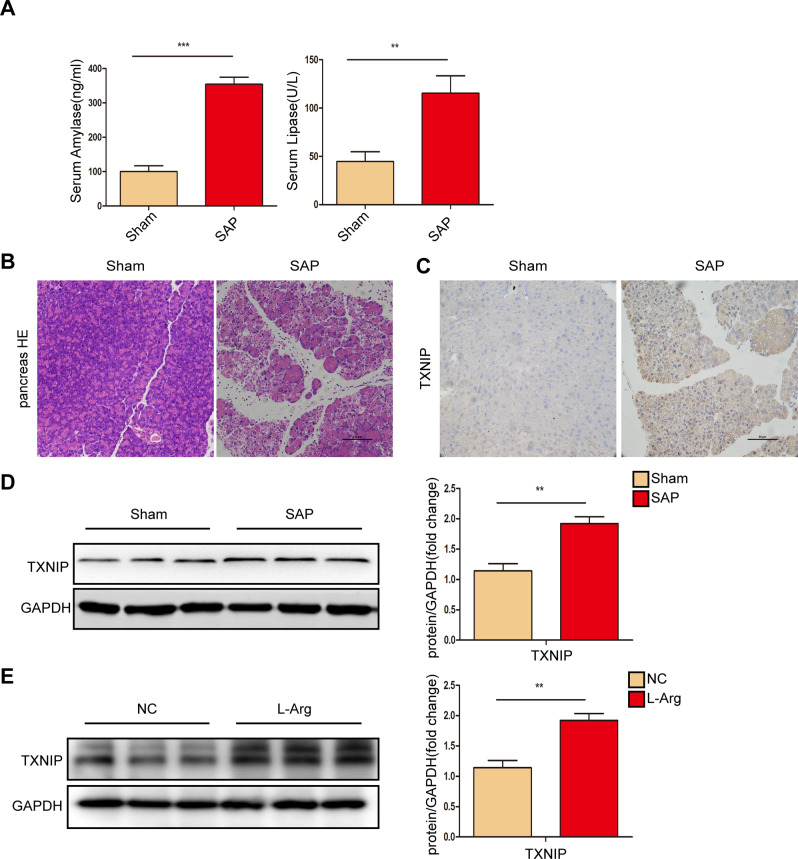
TXNIP expression was upregulated after SAP. **A** Amylase levels and lipase viability in serum of control and SAP groups (*n* = 6 /group). **B** Representative histological H&E staining images in pancreas of mice in different groups (*n* = 6/group). Scale bar = 100 µm. **C** Immunohistochemical staining of TXNIP in pancreas of mice in different groups (*n* = 6/group). Scale bar = 100 µm. **D** Protein levels of TXNIP in the pancreas of mice in Sham and SAP group (representative of three independent experiments). **E** Protein levels of TXNIP in AR42J cell in NC and L-Arg group (representative of three independent experiments). **D**, **E** used GAPDH as a loading control. **p < 0.01, ***p < 0.001.

To analyze the correlation between TXNIP and SAP, we first evaluated TXNIP expression after the induction of SAP in mice. Elevated levels of serum amylase and lipase are considered the most sensitive and specific markers of pancreatic acinar cell injury following SAP. As shown in Fig. 1A, the serum amylase and lipase levels of WT AP mice were significantly increased when compared with the sham group. Following the induction of SAP, there were significant increases in edema, inflammatory cell infiltration and necrosis of the pancreatic tissue (Fig. 1B); in addition, the expression of TXNIP in pancreatic tissue was significantly increased (Fig. 1C, D, Supplementary Fig. 1D). Furthermore, TXNIP expression was also higher in AR42J cells treated with L-Arg than in the untreated control (Fig. 1E, Supplementary Fig. 1E). These data indicated that after induction of SAP in mice, the expression of TXNIP was upregulated and that TXNIP was involved in SAP.

### TXNIP deletion or knockdown ameliorated SAP and SAP-related lung and kidney injury

**Fig. 2:**
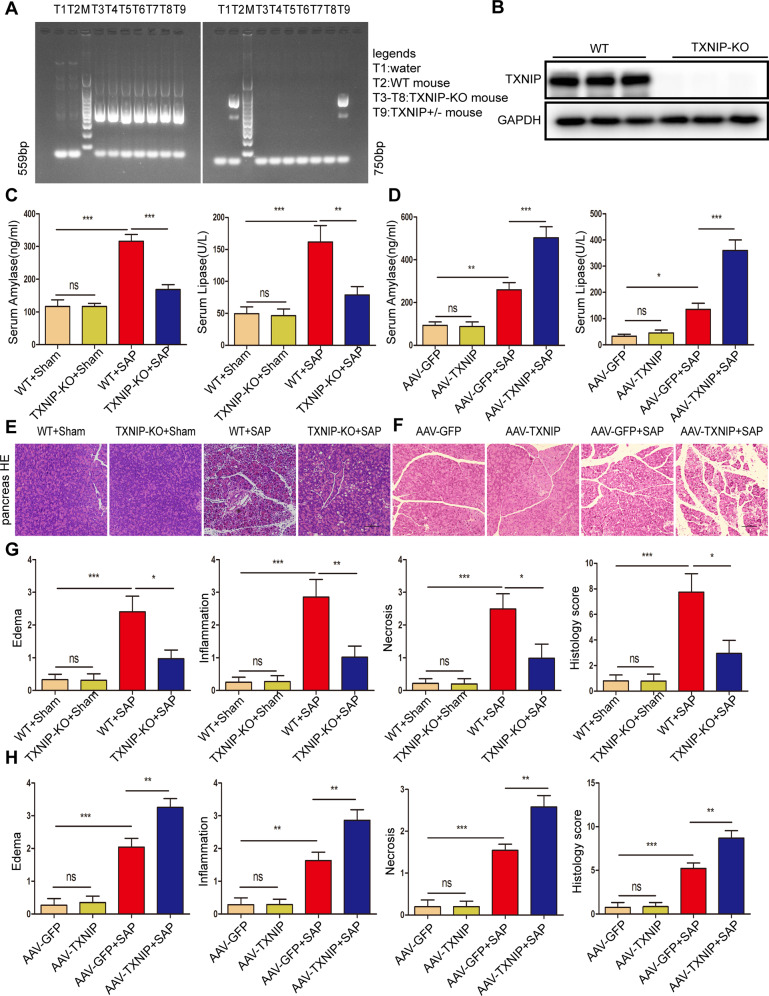
TXNIP deficiency improves SAP. **A** Mouse tail gene identification kit was used for gene identification, M was DNA Maker, T1 was water, T2 was WT mice, T3-T8 was TXNIP-KO mice, T9 were TXNIP heterozygous (TXNIP+/−) mice. **B** Protein levels of TXNIP in the pancreas of mice in WT and TXNIP-KO group (representative of three independent experiments). **C** Serum amylase levels and lipase viability in mice of the TXNIP-KO experimental group (*n* = 6/group). **D** Serum amylase levels and lipase viability in mice of the AAV-TXNIP experimental group (*n* = 6/group). **E** Representative histological H&E staining images in pancreas of mice in the TXNIP-KO experimental group (*n* = 6/group). Scale bar = 100 µm. **F** Representative histological H&E staining images in pancreas of mice in the AAV-TXNIP experimental group (*n* = 6/group). Scale bar = 100 µm **G** Statistics showing pathological injury in the pancreas of mice in the TXNIP-KO experimental group (*n* = 6/group). **H** Statistics showing pathological injury in the pancreas of mice in the AAV-TXNIP experimental group (*n* = 6/group). *p < 0.05, **p < 0.01, ***p < 0.001, ns *p* > 0.05.

We used TXNIP-KO mice to further study the role of TXNIP in SAP. Genomic DNA from mice tail tips were isolated for genotyping to confirm gene deletion (Fig. 2A, B, Supplementary Fig. 2B). After the induction of SAP, serum amylase levels and lipase activity were significantly reduced in the TXNIP-KO group when compared with the WT group; furthermore, the edema, inflammatory cell infiltration and necrosis of the pancreatic tissue were all significantly improved (Fig. 2C, E, G). After the induction of SAP, compared with AAV-GFP mice, serum amylase level and lipase activity were significantly increased in the AAV-TXNIP group; furthermore, edema, inflammatory cell infiltration, and necrosis in the pancreatic tissue were all significantly increased (Fig. 2D, F, H). These findings suggested that TXNIP deficiency improved SAP, whereas TXNIP overexpression aggravated the severity of SAP.

**Fig. 3:**
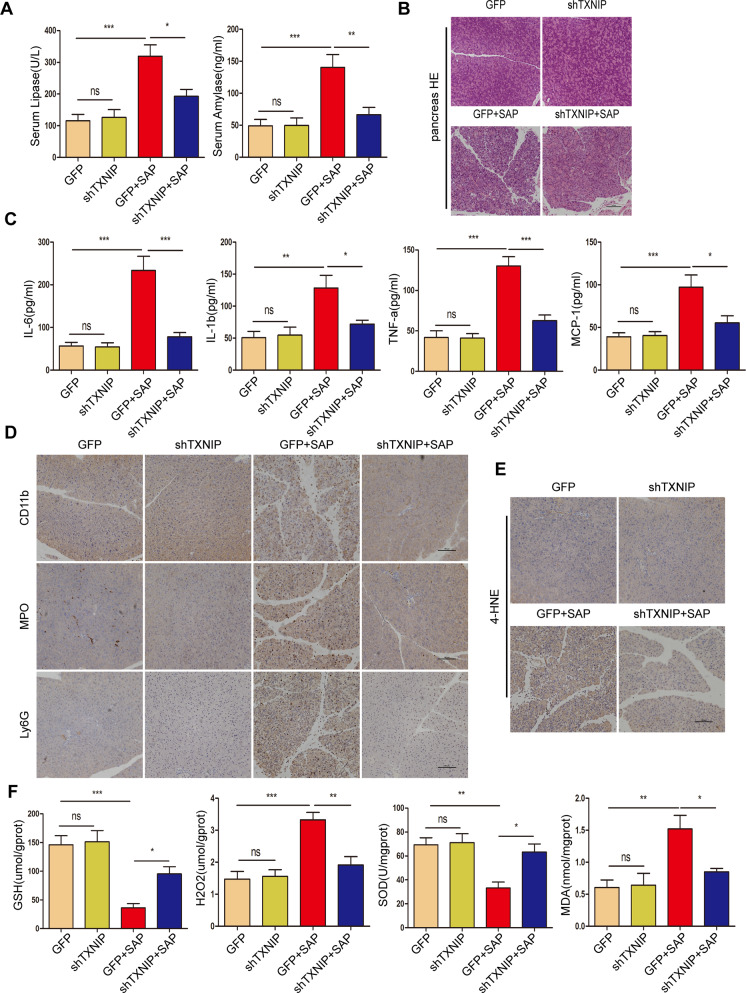
AAV-mediated TXNIP knockdown attenuated SAP in mice. **A** Serum amylase levels and lipase viability in mice of different groups (*n* = 6/group). **B** Representative histological H&E staining images in pancreas of mice in different groups (*n* = 6/group). Scale bar = 100 µm. **C** Serum levels of inflammatory factors (IL-6, IL-1β, TNF-α, and MCP-1) in mice of different groups (*n* = 6/group). **D**, **E** Immunohistochemical staining of CD11b, MPO, Ly6G, and 4-HNE in the pancreas of mice in different groups. Scale bar = 100 µm. **F** The levels of oxidative stress factors (GSH, H_2_O_2_, SOD, and MDA) detected by ELISA in the pancreas of mice in different groups (*n* = 6/group). *p < 0.05, **p < 0.01, ***p < 0.001, ns *p* > 0.05.

Next, we evaluated the therapeutic effects of AAV-mediated TXNIP knockdown on SAP. The serum amylase level and lipase activity were significantly decreased by shTXNIP after SAP (Fig. 3A). H&E staining and pathological scoring showed that edema, inflammatory cell infiltration and necrosis in the pancreatic tissue were significantly improved by shTXNIP after SAP (Figs. 3B, S1A). Next, we found that the levels of inflammatory factors (IL-6, IL-1β, TNF-α, and MCP-1) were significantly decreased by shTXNIP after SAP when compared with the control group (Fig. 3C). IHC staining of the pancreas also showed decreased levels of pancreatic CD11b, MPO, and Ly6G-positive inflammatory cells in response to shTXNIP after SAP (Fig. 3D), along with downregulated expression of 4-HNE (Fig. 3E). Moreover, ELISAs showed that the levels of oxidation-promoting molecules (H_2_O_2_ and MDA) in the pancreas were significantly reduced while the levels of antioxidant molecules (GSH and SOD) were increased by shTXNIP after SAP (Fig. 3F). Next, we performed histological analysis of the kidney and found that renal tubular necrosis, tubular dilation, loss of the brush border and cast formation were all reduced in the shTXNIP + SAP group when compared with the control group (Fig. S1B, C). Histological analysis revealed that edema and inflammatory cell infiltration in the lungs of shTXNIP mice after SAP were less severe when compared with those in the control group (Fig. S1D, E). Collectively, our data revealed that AAV-mediated TXNIP knockdown attenuated SAP in mice.

**Fig. 4:**
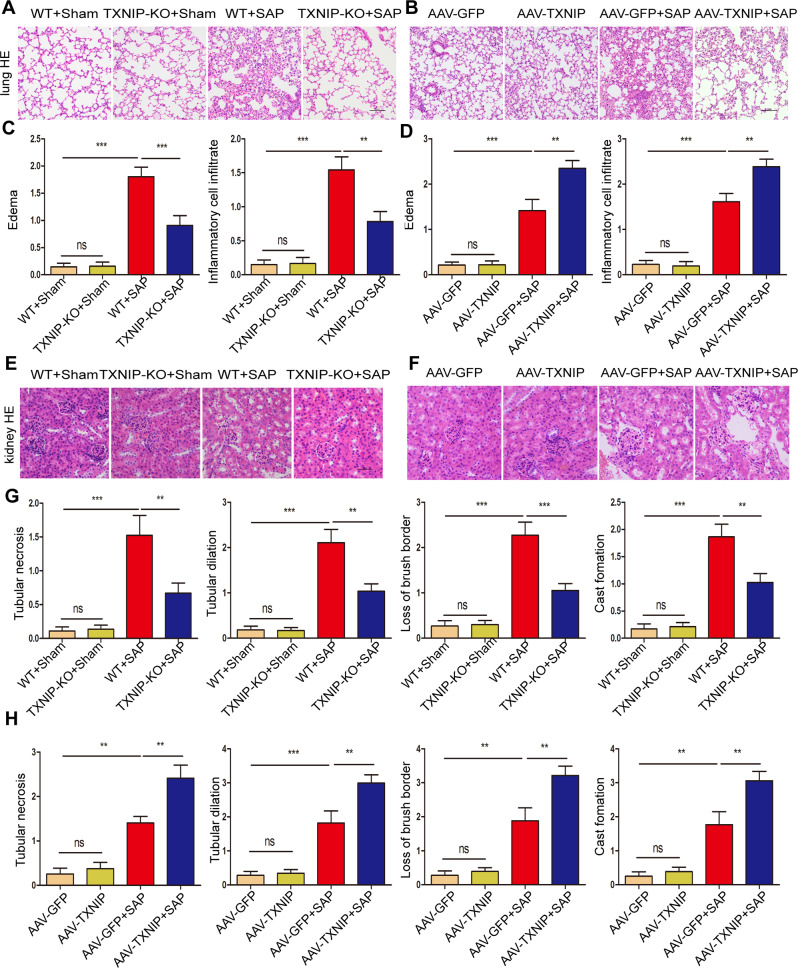
TXNIP deficiency ameliorates SAP-associated lung and kidney injury. **A** Representative histological H&E staining images in lung of mice in the TXNIP-KO experimental group (*n* = 6/group). Scale bar = 100 µm. **B** Representative histological H&E staining images in lung of mice in the AAV-TXNIP experimental group (n = 6/group). Scale bar = 100 µm. **C** Statistics showing pathological injury in the lung of mice in the TXNIP-KO experimental group (*n* = 6/group). **D** Statistics showing pathological injury in the lung of mice in the AAV-TXNIP experimental group (*n* = 6/group). **E** Representative histological H&E staining images in kidney of mice in the TXNIP-KO experimental group (*n* = 6/group). Scale bar = 50 µm. **F** Representative histological H&E staining images in kidney of mice in the AAV-TXNIP experimental group (*n* = 6/group). Scale bar = 50 µm. **G** Statistics showing pathological injury in the kidney of mice in the TXNIP-KO experimental group (*n* = 6/group). **H** Statistics showing pathological injury in the kidney of mice in the AAV-TXNIP experimental group (*n* = 6/group). **p < 0.01, ***p < 0.001, ns *p* > 0.05.

Next, we verified the effect of TXNIP on lung and kidney injury during SAP. After the induction of SAP and compared with the WT group, the lung tissue of the TXNIP-KO group showed less edema and inflammatory cell infiltration (Fig. 4A, C); furthermore, there were clear reductions in tubular necrosis, tubular dilation, loss of brush border and cast formation (Fig. 4E, G). Following the induction of SAP and compared with AAV-GFP group, the lung tissue of AAV-TXNIP mice showed significantly increased symptoms of edema and inflammatory cell infiltration (Fig. 4B, D); in addition, tubular necrosis, tubular dilation, and the loss of brush borders and cast formation were significantly increased (Fig. 4F, H). These results showed that TXNIP deficiency protected against SAP-associated lung and kidney injury.

### TXNIP regulated the inflammatory response and mediated oxidative stress in the process of SAP

**Fig. 5:**
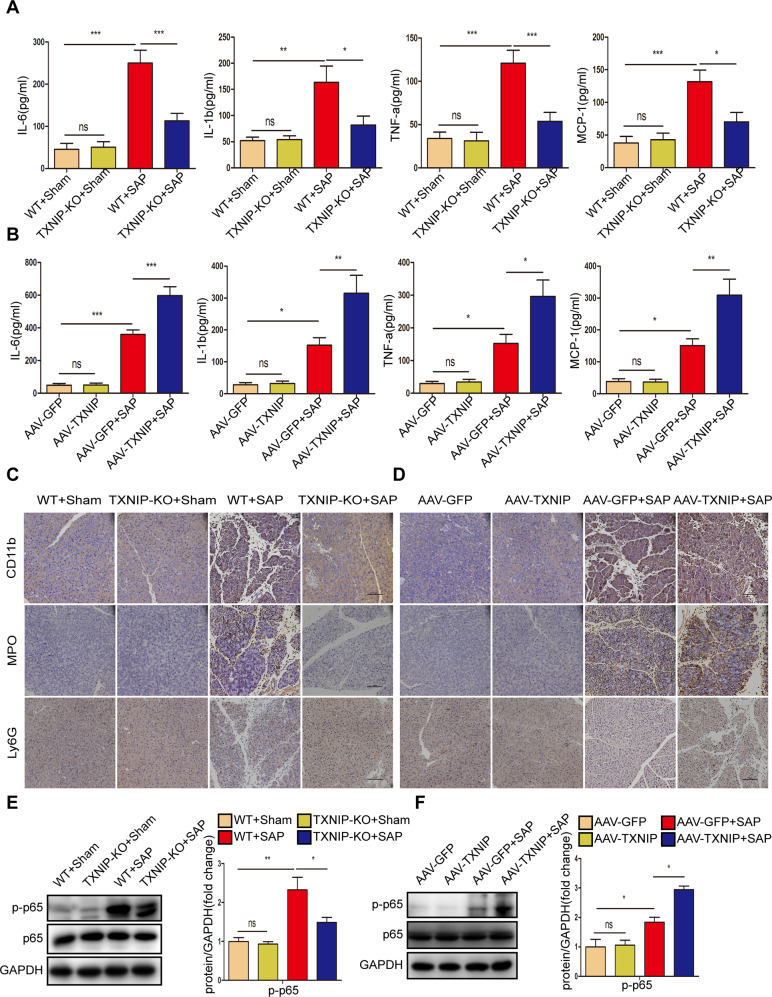
TXNIP regulates inflammatory response in the process of SAP. **A** Serum levels of inflammatory factors (IL-6, IL-1β, TNF-α, and MCP-1) in mice of the TXNIP-KO experimental group (*n* = 6/group). **B** Serum levels of inflammatory factors (IL-6, IL-1β, TNF-α, and MCP-1) in mice of the AAV-TXNIP experimental group (*n* = 6/group). **C** Representative immunohistochemical staining of CD11b, MPO, and Ly6G in the pancreas of mice in the TXNIP-KO experimental group (*n* = 6/group). Scale bar=100 µm. **D** Representative immunohistochemical staining of CD11b, MPO, and Ly6G in the pancreas of mice in the AAV-TXNIP experimental group (*n* = 6/group). Scale bar = 100 µm. **E** Protein levels of p65 and p-p65 in the pancreas of mice in the TXNIP-KO experimental group (representative of three independent experiments). **F** Protein levels of p65 and p-p65 in the pancreas of mice in the AAV-TXNIP experimental group (representative of three independent experiments). **E**, **F** used GAPDH as a loading control. *p < 0.05, **p < 0.01, ***p < 0.001, ns *p* > 0.05.

Inflammatory factors and chemokines play a major role in the development of pancreatitis [2]. After the induction of SAP, TXNIP deletion significantly reduced the expression levels of inflammatory factors (IL-6, IL-1β, TNF-α, and MCP-1); when TXNIP was overexpressed, the expression levels of these inflammatory factors were significantly increased (Fig. 5A, B). Following the induction of SAP, we performed IHC staining for inflammatory cell markers (CD11b, MPO, and Ly6G). As shown in Fig. 5C, D, the number of CD11b-, MPO-, and Ly6G-positive inflammatory cells were significantly reduced in the absence of TXNIP; in contrast, the number of MPO- and Ly6G-positive inflammatory cells were significantly elevated. Furthermore, TXNIP depletion reduced the expression of phosphorylated p65 after the induction of SAP, whereas TXNIP overexpression increased the level of phosphorylated p65 (Fig. 5E, F, Supplementary Fig. 5E, F). These data suggested that TXNIP modulates the inflammatory response in SAP mice.

**Fig. 6:**
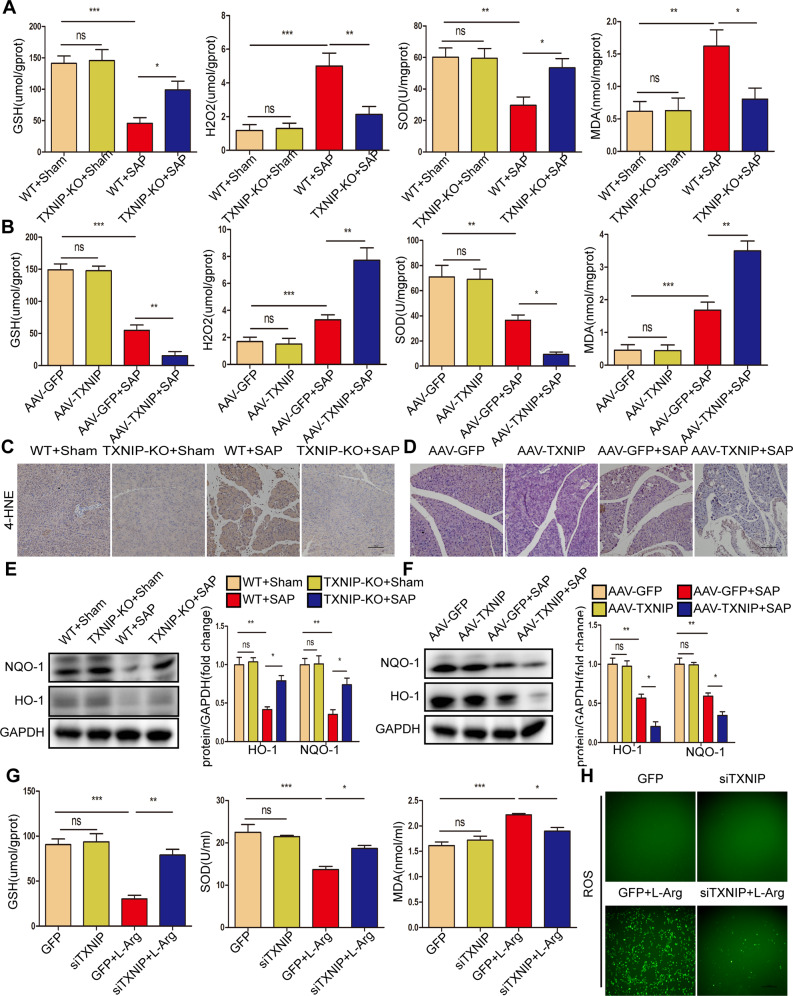
TXNIP mediates oxidative stress during SAP. **A** The levels of oxidative stress factors (GSH, H_2_O_2_, SOD, and MDA) detected by ELISA in the pancreas of mice in the TXNIP-KO experimental group (*n* = 6/group). **B** The levels of oxidative stress factors (GSH, H_2_O_2_, SOD, and MDA) detected by ELISA in the pancreas of mice in the AAV-TXNIP experimental group (*n* = 6/group). **C** Representative immunohistochemical staining of 4-HNE in the pancreas of mice in the TXNIP-KO experimental group (*n* = 6/group). Scale bar=100 µm. **D** Representative immunohistochemical staining of 4-HNE in the pancreas of mice in the AAV-TXNIP experimental group (*n* = 6/group). Scale bar = 100 µm. **E** Protein levels of HO-1 and NQO-1 in the pancreas of mice in the TXNIP-KO experimental group (representative of three independent experiments). **F** Protein levels of HO-1 and NQO-1 in the pancreas of mice in the AAV-TXNIP experimental group (representative of three independent experiments). **E**, **F** used GAPDH as a loading control. **G** The levels of oxidative stress factors (GSH, SOD, and MDA) detected by ELISA in AR42J cells of different groups (*n* = 6/group). **H** Representative images showing ROS in the AR42J cells in different groups (*n* = 6/group). Scale bar = 100 µm. *p < 0.05, **p < 0.01, ***p < 0.001, ns *p* > 0.05.

During SAP, oxidative stress not only acts as a damaging factor but also triggers proinflammatory cytokine production [23]. Therefore, we further explored whether TXNIP regulates oxidative stress during SAP. After the induction of SAP, the oxidation-promoting molecules (H_2_O_2_ and MDA) in the pancreas were significantly reduced in TXNIP-KO mice, but significantly increased in AAV-TXNIP mice; in contrast, the expression levels of antioxidant molecules (GSH and SOD) showed an opposite trend (Fig. 6A, B). The activity of the peroxidation product 4-HNE was significantly reduced in TXNIP-KO mice and increased in AAV-TXNIP mice (Fig. 6C, D). Furthermore, after the induction of SAP, the antioxidant proteins HO-1 and NQO-1 were highly expressed in TXNIP-KO mice but were only expressed at low levels in AAV-TXNIP mice (Fig. 6E, F, Supplementary Fig. 6E, F). To further determine the effect of TXNIP on pancreatic acinar cells, we treated AR42J cells with L-Arg. Consistent with the in vivo results, TXNIP knockdown significantly increased the levels of GSH and SOD and reduced the levels of MDA (Fig. 6G). In addition, TXNIP knockdown significantly suppressed ROS production after L-Arg treatment (Fig. 6H), decreased ACSL4 protein expression, but increased GPX4 expression (Fig. S2). These results suggest that TXNIP mediates oxidative stress during SAP.

### TXNIP deficiency inhibits ASK1 activation and downstream JNK/p38 signaling during SAP

**Fig. 7:**
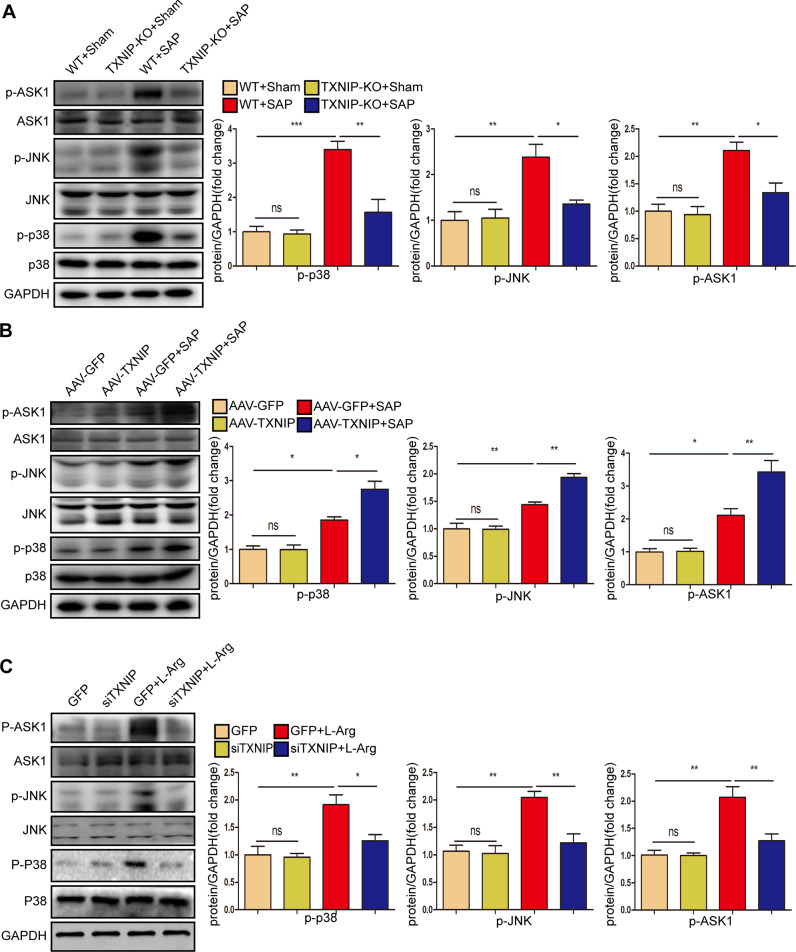
TXNIP deficiency inhibits ASK1 activation and downstream JNK/p38 signaling during SAP. **A** Western blot analysis of the total and phosphorylated protein expression levels of p38 JNK and ASK1 in the pancreas of mice in the TXNIP-KO experimental group (representative of three independent experiments). **B** Western blot analysis of the total and phosphorylated protein expression levels of p38 JNK and ASK1 in the pancreas of mice in the AAV-TXNIP experimental group (representative of three independent experiments). **C** Western blot analysis of the total and phosphorylated protein expression levels of p38 JNK and ASK1 in the AR42J cells of different groups (representative of three independent experiments). **A**–**C** used GAPDH as a loading control. *p < 0.05, **p < 0.01, ***p < 0.001, ns *p* > 0.05.

TXNIP promoted the inflammatory response and oxidative stress in SAP; therefore, we further explored the potential mechanism of TXNIP in SAP. Mitogen-activated protein kinase (MAPK) signaling is essential for the occurrence and maintenance of pancreatitis, and inhibition of this signaling pathway is known to reduce inflammation and fibrosis [24, 25]. Therefore, we next investigated whether TXNIP regulates the MAPK signaling pathway in the process of SAP. We found that the phosphorylation of p38, JNK, and ASK1 was activated after SAP, but was decreased in TXNIP-KO mice after SAP (Fig. 7A, Supplementary Fig. 7A). We also found the opposite trend in AAV-TXNIP mice (Fig. 7B, Supplementary Fig. 7B). Furthermore, TXNIP knockdown suppressed activated p-p38, p-JNK, and p-ASK1 in AR42J cells when stimulated by L-Arg (Fig. 7C, Supplementary Fig. 7C). Collectively, these results suggested that TXNIP deletion inhibited ASK1 activation and the downstream JNK/p38 signaling pathway during SAP.

### TXNIP affected inflammation and oxidative stress during SAP by regulating ASK1

**Fig. 8:**
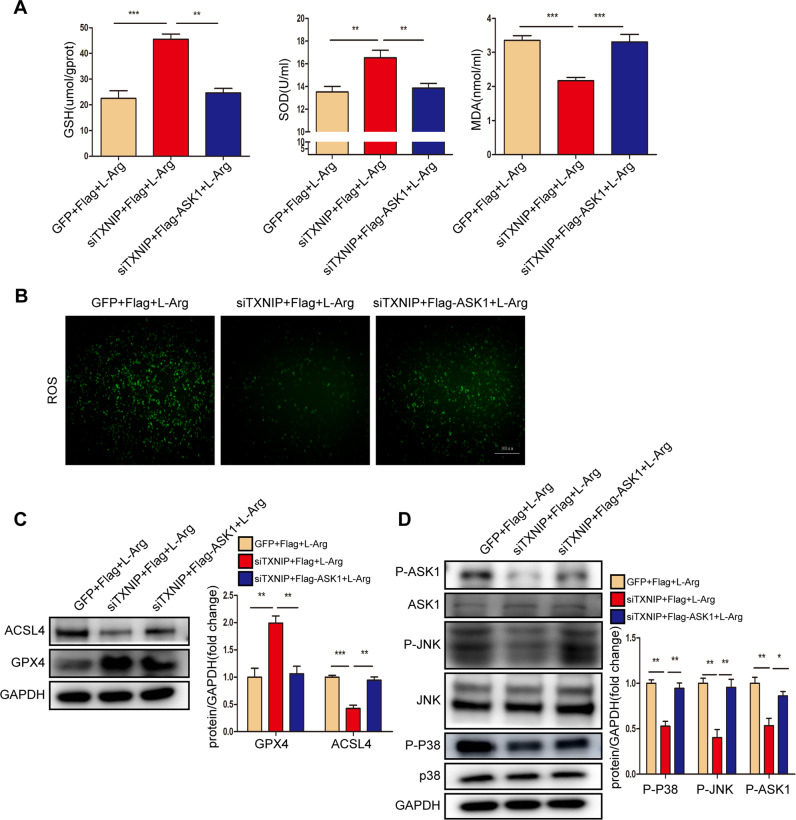
ASK1 accelerates pancreatic acinar cells injury after L-Arg treatment. **A** The levels of oxidative stress factors (GSH, SOD, and MDA) detected by ELISA in AR42J cells induced by L-Arg of different groups (*n* = 6/group). **B** Representative images showing ROS in the AR42J cells induced by L-Arg of different groups (*n* = 6/group). Scale bar = 100 µm. **C** Protein levels of GPX4 and ACSL4 in AR42J cells induced by L-Arg of different groups (representative of three independent experiments). **D** Western blot analysis of the total and phosphorylated protein expression levels of p38 JNK and ASK1 in the AR42J cells induced by L-Arg of different groups (representative of three independent experiments). **C**, **D** used GAPDH as a loading control. *p < 0.05, **p < 0.01, ***p < 0.001.

ASK1 mediates the effects of TXNIP on SAP in vivo; therefore, we next determined whether ASK1 mediates the effects of TXNIP on L-Arg-stimulated AR42J cells. The results showed that ASK1 overexpression promoted the production of ROS, significantly decreased the expression of antioxidant molecules (GSH and SOD) and increased the expression of the oxidation-promoting molecule MDA in AR42J cells (Fig. 8A, B). Furthermore, ASK1 overexpression decreased GPX4 protein expression but promoted ACSL4 expression and p-p38, p-JNK, and p-ASK1 production in AR42J cells (Fig. 8C, D, Supplementary Fig. 8C, D). Thus, ASK1 accelerated pancreatic acinar cell damage after L-Arg treatment.

**Fig. 9:**
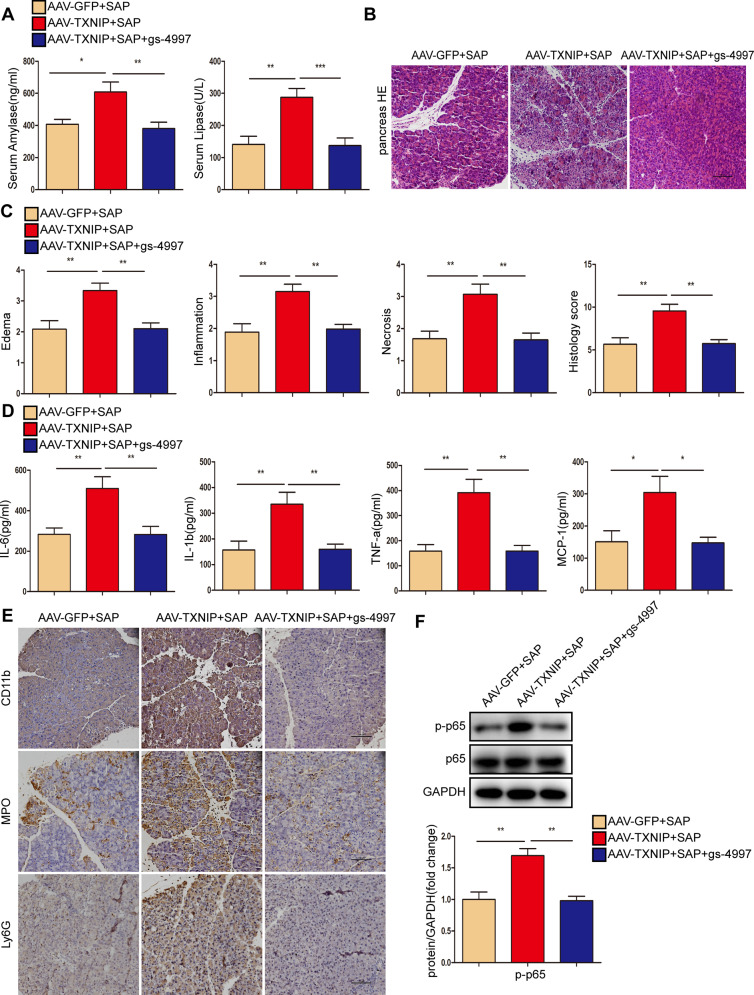
ASK1 mediated the effect of TXNIP on inflammation during SAP. **A** Serum amylase levels and lipase viability in mice of different groups (*n* = 6/group). **B** Representative histological H&E staining images in pancreas of mice in different groups (*n* = 6/group). Scale bar = 100 µm. **C** Statistics showing pathological injury in the pancreas of mice in different groups (*n* = 6/group). **D** Serum levels of inflammatory factors (IL-6, IL-1β, TNF-α, and MCP-1) in mice of different groups (*n* = 6/group). **E** Representative immunohistochemical staining of CD11b, MPO, and Ly6G for mouse pancreas in different groups (*n* = 6/group). Scale bar = 100 µm. **F** Protein levels of p65 and p-p65 in the pancreas of mice in different groups (representative of three independent experiments). GAPDH was used as a loading control. *p < 0.05, **p < 0.01, ***p < 0.001.

Next, we used the ASK1 inhibitor gs-4997 to assess the role of ASK1 in TXNIP-mediated SAP in vivo. Compared with the AAV-TXNIP + SAP group, gs-4997 largely abolished the increase in serum amylase level and lipase activity in mice after SAP, alleviated pancreatic tissue edema, inflammation, and necrosis, and reduced the expression levels of inflammatory factors (Fig. 9A–D). Furthermore, gs-4997 treatment reduced the number of CD11b-, MPO-, and Ly6G-positive inflammatory cells in pancreatic tissue and reduced the expression of p-p65 after the induction of SAP (Fig. 9E, F, Supplementary Fig. 9F). These results demonstrated that ASK1 mediates the effects of TXNIP on inflammation during SAP.

**Fig. 10:**
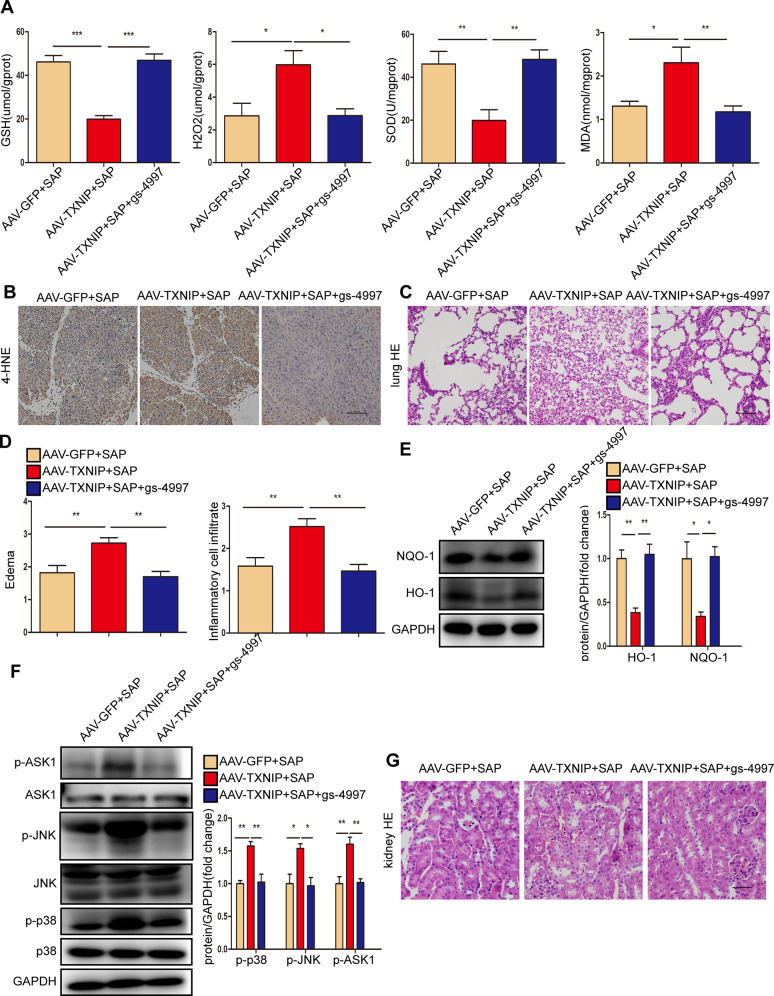
ASK1 mediated the effect of TXNIP on oxidative stress during SAP. **A** The levels of oxidative stress factors (GSH, H_2_O_2_, SOD, and MDA) detected by ELISA in the pancreas of mice in different groups (*n* = 6/group). **B** Representative immunohistochemical staining of 4-HNE in the pancreas of mice in different groups (*n* = 6/group). Scale bar = 100 µm. **C** Representative histological H&E staining images in lung of mice in different groups (*n* = 6/group). Scale bar = 100 µm. **D** Statistics showing pathological injury in the lung of mice in different groups (*n* = 6/group). **E** Protein levels of HO-1 and NQO-1 in the pancreas of mice in different groups (representative of three independent experiments). **F** Western blot analysis of the total and phosphorylated protein expression levels of p38 JNK and ASK1 in the pancreas of mice in different groups (representative of three independent experiments). **E**, **F** used GAPDH as a loading control. **G** Representative histological H&E staining images in kidney of mice in different groups (*n* = 6/group). Scale bar = 50 µm. *p < 0.05, **p < 0.01, ***p < 0.001.

Next, we verified the role of ASK1 in TXNIP-mediated oxidative stress during SAP. Compared with the AAV-TXNIP + SAP group, gs-4997 treatment increased the expression of antioxidant molecules (GSH and SOD) and antioxidant proteins (HO-1 and NQO-1) in pancreatic tissue but also promoted oxidation. The expression levels of H_2_O_2_ and MDA, as well as the peroxidation product 4-HNE, were significantly reduced (Fig. 10A, B, E, Supplementary Fig. 10E). In addition, the phosphorylation levels of p38, JNK, and ASK1 were decreased after gs-4997 treatment (Fig. 10F, Supplementary Fig. 10F). Treatment with gs-4997 also reduced lung tissue edema and inflammatory cell infiltration, and reduced tubular necrosis, tubular dilation, brush border loss and cast formation in renal tissue, thereby attenuating SAP-related lung and kidney injury (Figs. 10C, D, G, S3).

## Discussion

**Fig. 11:**
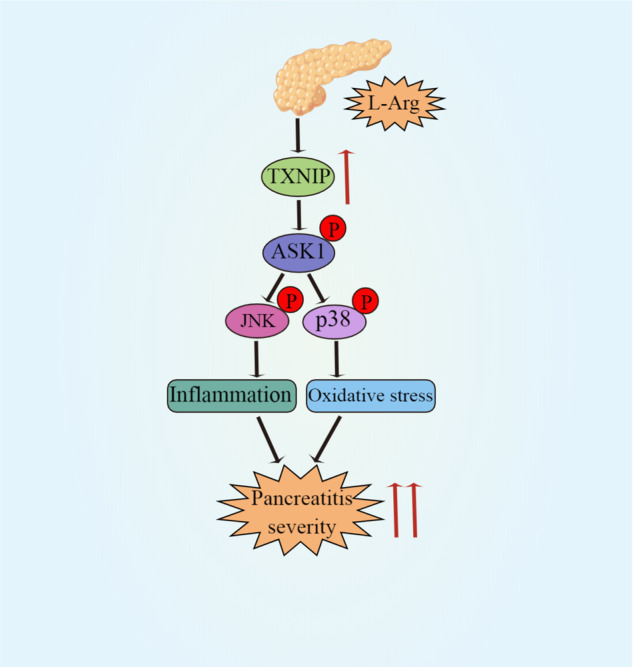
Schematic diagram of the mechanism of TXNIP action in L-Arg induced SAP. TXNIP mediates the inflammatory response and oxidative stress during L-Arg-induced SAP by regulating the ASK1-JNK/p38 signaling pathway.

AP is widely recognized to be a digestive disease associated with inflammation [26]. Mild acute pancreatitis usually involves only edema and inflammation of the pancreas with less necrosis and bleeding. When SAP develops, it is often accompanied by bleeding and necrosis of tissue lesions. In severe cases, systemic inflammation and failure of the surrounding organs will occur [27, 28]. The inflammatory response and oxidative stress play an indispensable role in SAP [20, 22]. TXNIP is a multi-functional protein that acts as a signal conductor of the oxidative stress response [29]. Importantly, TXNIP is vital for inflammation and oxidative stress [30]. In this study, TXNIP expression was significantly upregulated after SAP. TXNIP-KO, AAV-TXNIP, and shTXNIP mice revealed the damaging effect of TXNIP in the pancreas after SAP. Further molecular analyses demonstrated that TXNIP overexpression activated ASK1, thereby promoting activation of the ASK1-dependent JNK/p38 signaling pathway. Overall, our study suggests that TXNIP is a major mediator of pancreatic injury in SAP and plays an important role in pancreatic pathology (Fig. 11).

Inflammation is considered as a marker of SAP development. The inflammatory response removes cells damaged by injury and infection and then initiates tissue repair. Under conditions of stress, cytokines and inflammatory mediators are generally released by infiltrating inflammatory cells. PSCs are important players in AP development [31]. The damage of pancreatic tissue triggers the activation of PSC, and prompts immune cells and pancreatic acinar cells to secrete inflammatory mediators, which promote the development of AP [32, 33]. PSC activation is increasingly recognized as important in pancreatitis, involving many signaling pathways, including ERK, NF-κB, and Ca signaling [8, 34–36]. Activated PSCs also secrete the neutrophil chemokine IL-8 and macrophage chemoattractant protein-1 (MCP-1) and express intracellular adhesion molecule-1 (ICAM-1) [36, 37], suggesting that activated PSCs may be involved in exacerbating pancreatic inflammation by recruiting inflammatory cells. Studies have shown that pancreatic acinar cells secrete various inflammatory cytokines in response to the stimulation of pancreatitis [16, 20]. The inflammatory response during SAP is characterized by the induction of a cascade of proinflammatory mediators which culminates in the recruitment of macrophages and neutrophils to the inflamed pancreas, thus leading to pancreatic acinar cell injury [38]. Under conditions of stress, infiltrating inflammatory cells generally respond to SAP stimulation through the release of proinflammatory factors such as IL-6, IL-1β, TNF-α, and MCP-1 [12, 39]. TXNIP has been reported to play an important role in regulating the inflammatory response and deletion of TXNIP has been shown to attenuate the inflammatory response and fibrosis in kidney disease [40]. Consistent with these results, TXNIP deficiency reduced the levels of proinflammatory factors such as IL-6, IL-1β, TNF-α, and MCP-1. In addition, the deletion of TXNIP also reduced the number of infiltrating CD11b-, MPO-, and Ly6G-positive inflammatory cells, whereas TXNIP overexpression had the opposite effect. Thus, TXNIP regulates the inflammatory response during SAP.

The imbalance between oxidative and antioxidant systems leads to oxidative stress that has been recognized as an initial step in many pathological processes [41]. Oxidative stress plays a major role in the development of SAP. Brookes et al. previously suggested that calcium signaling and ROS are correlated and apoptosis occurs when calcium-dependent ROS are excessively elevated in acinar cells [42, 43]. Mitochondria supply ATP for normal activities of the body and are also the main source of ROS in cells. Mitochondrial damage and ATP depletion play a central role in the development of SAP [44]. In the present study, SAP and the treatment of pancreatic acinar cells with L-Arg caused oxidative stress, thus resulting in reduced levels of GSH and SOD, and increased levels of MDA, ROS, and H_2_O_2_. The imbalance between the oxidant and antioxidant systems, and the increase of oxidation damage in tissues and cells, results in the body incurring damage. TXNIP plays a major role in regulating inflammation and oxidative stress by regulating the intracellular environmental balance, binding and inhibiting antioxidants and mercaptans, and reducing the activity of TRX, thus leading to oxidative stress and aseptic inflammatory responses, thereby affecting cell differentiation, metabolism, and senescence [14, 29]. RNA interference of TXNIP has been shown to alleviate oxidative stress induced by high levels of uric acid [45]. The production of the superoxide anion, hydroxyl, peroxy, alkoxy, nitric oxide free radicals, and ROS causes cellular damage, thus leading to an inflammatory reaction [46]. Free radicals, cell damage, and inflammation interact and influence each other during the entire process of oxidative stress. In this study, we consistently found that TXNIP affected the balance between oxidation and antioxidation. In animal experiments, TXNIP knockout increased the levels of GSH and SOD, decreased the levels of MDA and H_2_O_2_, downregulated 4-HNE, and upregulated HO-1 and NQO-1 to alleviate oxidative stress during SAP. In cell experiments, the downregulation of TXNIP increased the levels of GSH and SOD, decreased MDA and ROS, downregulated ACSL4, and upregulated GPX4 to reduce oxidative stress in L-Arg-induced AR42J cells. However, our in vivo experiments of TXNIP overexpression and our in vitro experiments of ASK1 overexpression showed the opposite results. Collectively, our study demonstrated that TXNIP regulates oxidative stress during SAP.

During the process of SAP development, not only the pancreas itself but also the associated distant organs, such as the lungs and kidneys, are also challenged by damage caused by inflammation and oxidative stress [47]. Acute lung injury is a major extra-pancreas complication. Neutrophils are the first immune cells recruited to the inflammatory site of the lung and play a major role in SAP-related lung injury [48]. In this study, we found that the deletion or downregulation of TXNIP in L-Arg-induced SAP reduced pulmonary inflammation and edema, whereas the overexpression of TXNIP further aggravated the degree of inflammatory cell infiltration and edema. In addition, acute kidney injury is a common complication of SAP with a poor prognosis. The infiltration of inflammatory cells, along with the release of proinflammatory factors, act on endothelial cells, glomeruli, and tubular capillaries, thus leading to renal ischemia and necrosis [49, 50]. In our previous study, macrophage mobility inhibitory factor inhibitor therapy ameliorated SAP-associated acute kidney injury by inhibiting the NLRP3 inflammasome signaling pathway [51]. The results of the present study suggest that TXNIP deletion or downregulation improves SAP-related renal tubular necrosis, tubular dilation, loss of the brush border, and cast formation, whereas TXNIP overexpression aggravates pathological changes in the kidney. Thus, TXNIP regulates SAP-related lung and kidney injuries.

ASK1 is a member of the mitogen-activated protein kinase kinase kinase family, including downstream MAPKs, JNKs, and p38MAPKs and plays major biological roles in SAP progression, including the inflammatory response, DNA damage, and apoptosis [52–55]. Following various stresses (e.g., ROS, tumor necrosis factors, microtubule interferers, and cancer chemotherapy drugs), the activated form of ASK1 activates mitogen-activated protein kinase kinase; downstream, JNK and p38 shuttle from the cytoplasm to the nucleus and catalyze the phosphorylation of various proteins and transcription factors [21]. This can lead to inflammation and oxidative stress. The ASK1-dependent JNK/p38 signaling pathway is associated with various human diseases, such as cardiovascular diseases, diabetes, and liver diseases [56–58]. Studies have shown that ceramide activates ASK1 through the upregulation of TXNIP expression [59]. Consistent with these results, we observed that TXNIP overexpression activated ASK1, leading to a significant increase in the phosphorylation levels of JNK and p38 proteins, whereas TXNIP knockout or downregulation showed the opposite trend. Studies have found that the binding of N-acetylgalactosaminyltransferase-4 to ASK1 inhibits its N-terminal dimerization and subsequent phosphorylation, thus leading to strong inactivation of downstream JNK/p38 signaling [60]. Moreover, the therapeutic use of ASK1 inhibitors has been extensively described in several reviews [61–63]. Selonsertib (gs-4997) is a selective ASK1 inhibitor that reduces metabolic parameters, inflammation, and fibrosis in multi-drug resistance and non-alcoholic hepatitis [64, 65]. In this study, the levels of proinflammatory factors (IL-6, IL-1β, TNF-α, and MCP-1) were decreased significantly, the infiltration of CD11b-, MPO-, Ly6G-, and 4-HNE-positive inflammatory cells was decreased, the levels of GSH and SOD were upregulated, and the levels of H_2_O_2_ and MDA were downregulated after gs-4997 treatment. These data indicated that gs-4997 improved SAP by inhibiting ASK1 activation. Further molecular analyses also showed that the phosphorylation levels of ASK1, JNK, and p38 proteins were decreased. In cell experiments, ASK1 overexpression increased oxidative stress and aggravated pancreatic acinar cell damage. Thus, ASK1 mediates the effects of TXNIP on the inflammatory response and oxidative stress in SAP and L-Arg-treated AR42J cells.

In conclusion, our study confirms TXNIP as a novel regulator of SAP. TXNIP induces the phosphorylation of the JNK/p38 signaling pathway by activating ASK1, thus leading to an inflammatory response and oxidative stress. These findings broaden our understanding of the regulatory role TXNIP in the pancreas. Therefore, the ASK1-JNK/p38 pathway, which is regulated by TXNIP, may be a major mechanism of SAP; the inhibition of TXNIP expression in acinar cells may provide a therapeutic strategy for SAP in the clinic. This study also had some limitations that need to be considered. First, this study was limited to animals and did not involve clinical SAP patients. Furthermore, the potential mechanisms by which TXNIP can regulate pancreatitis through ASK1-JNK/p38 requires further study. We hope that relevant experiments can be carried out in the clinic in the future to facilitate the clinical treatment of SAP.

## Supplementary information

Supplemental Material--Full and uncropped western blots

Supplementary material

Reproducibility checklist

## Data Availability

The datasets generated or analyzed during this study are available from the corresponding authors on reasonable request.
