# Supplementary figures and images for: Thioredoxin-interacting protein deficiency protects against severe acute pancreatitis by suppressing apoptosis signal-regulating kinase 1
Source: Cell Death Dis. 2022 Oct 31;13(10):914. doi: 10.1038/s41419-022-05355-x (PMC9622726; doi:10.1038/s41419-022-05355-x)

**Fig.1.**

**E**

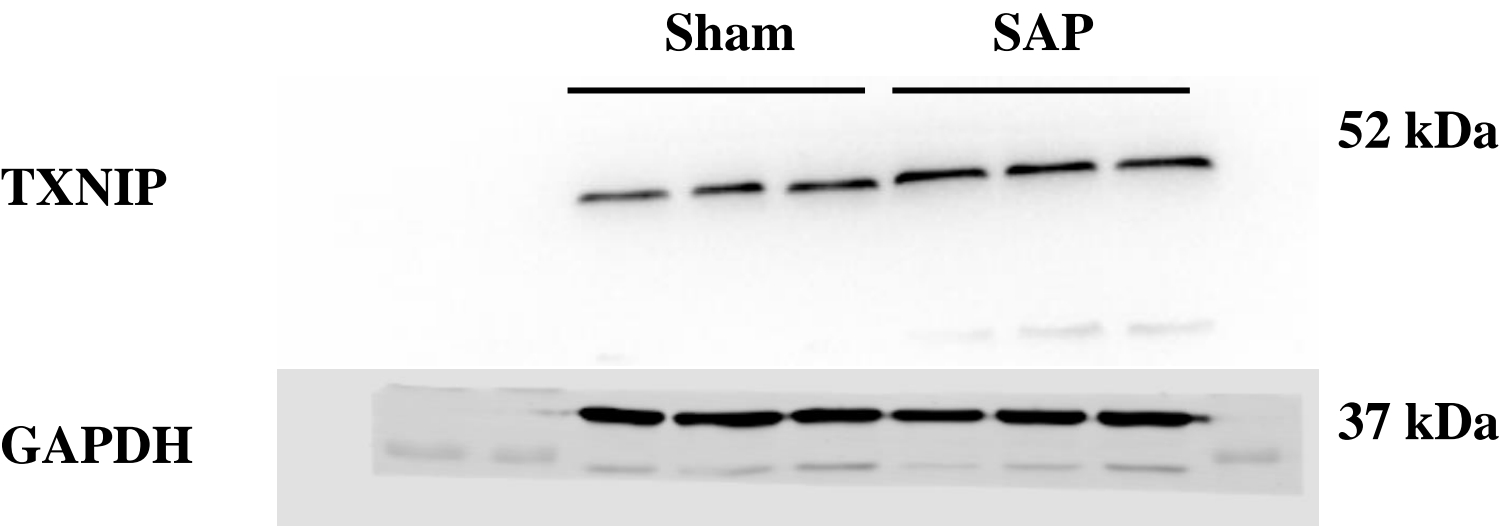

**F**

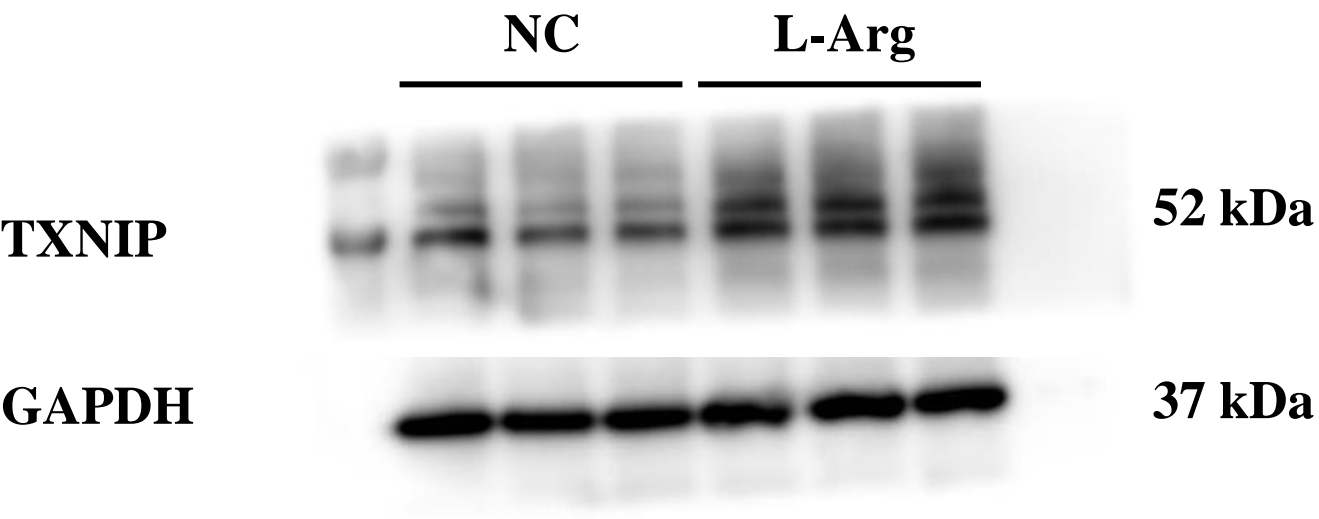

**Fig. 2.**

# B

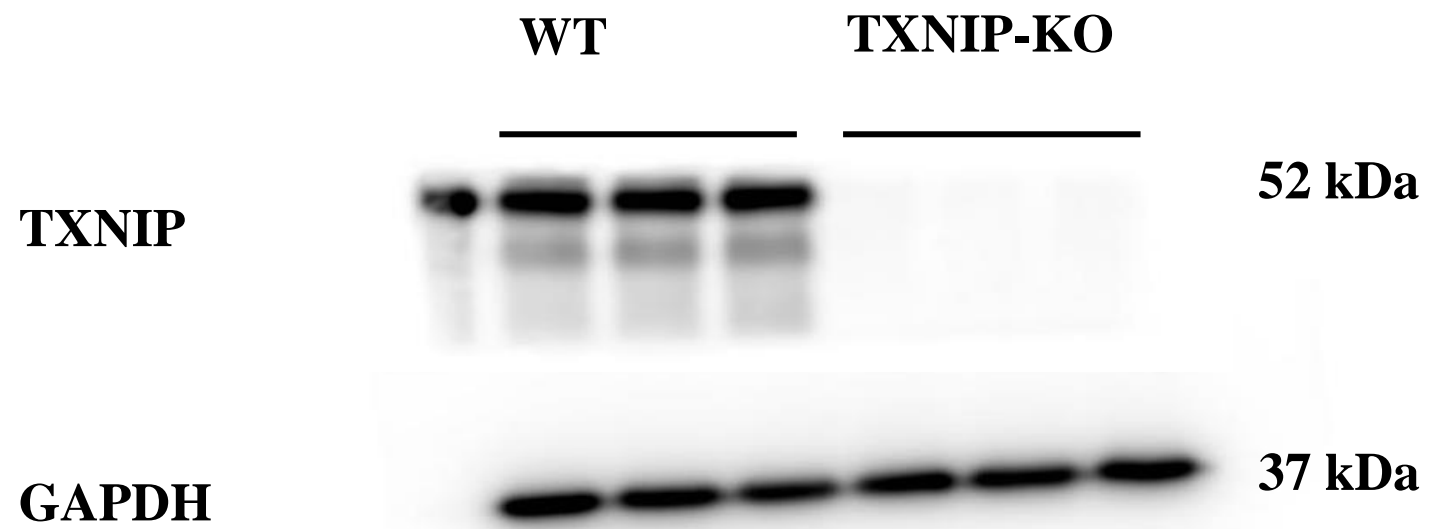

**Fig. 5.**

**E**

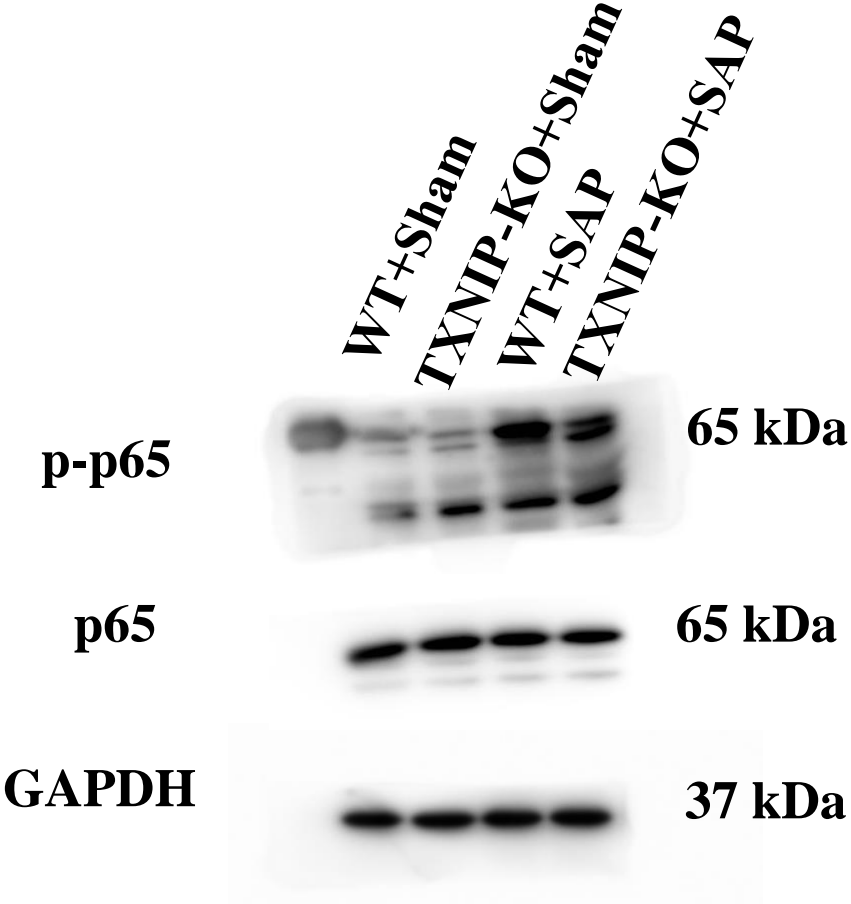

**F**

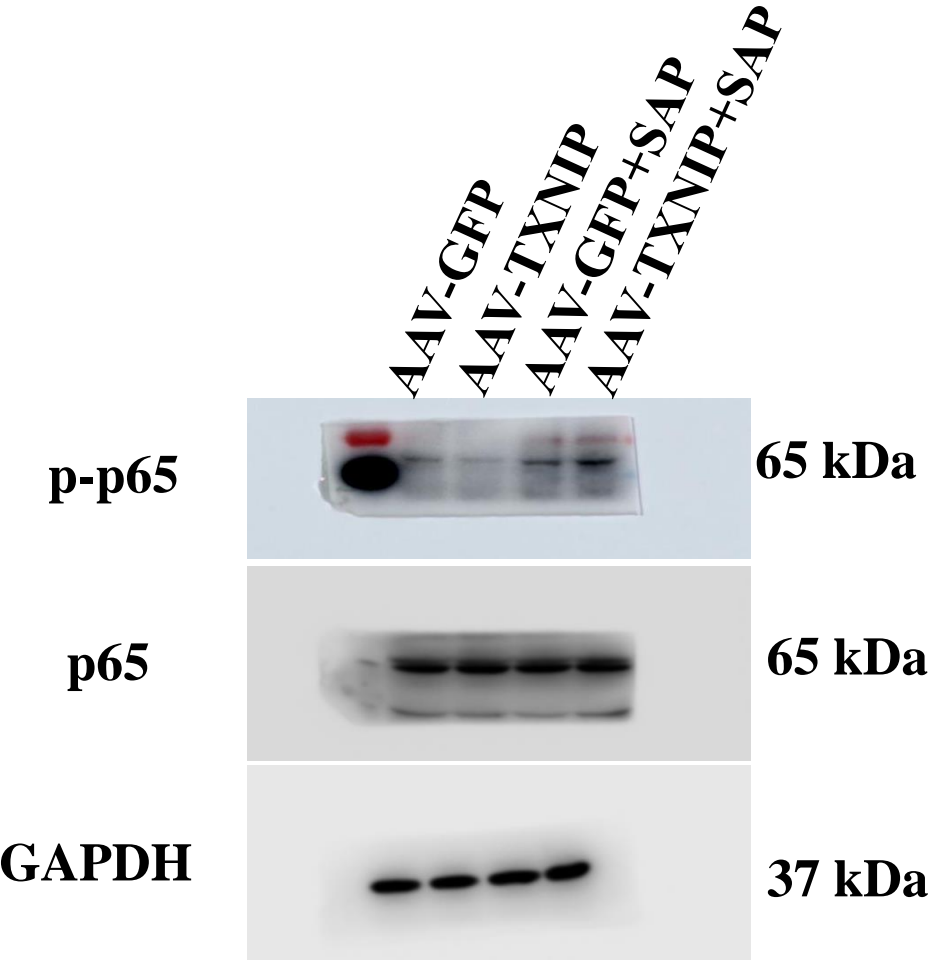

**Fig. 6.**

**E**

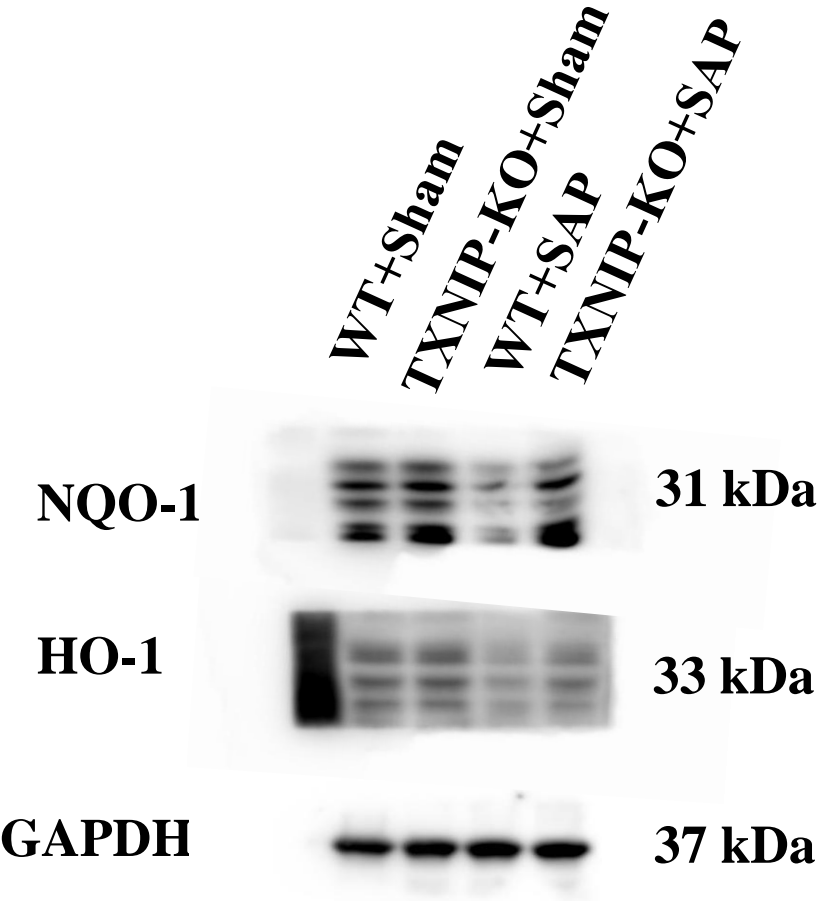

**F**

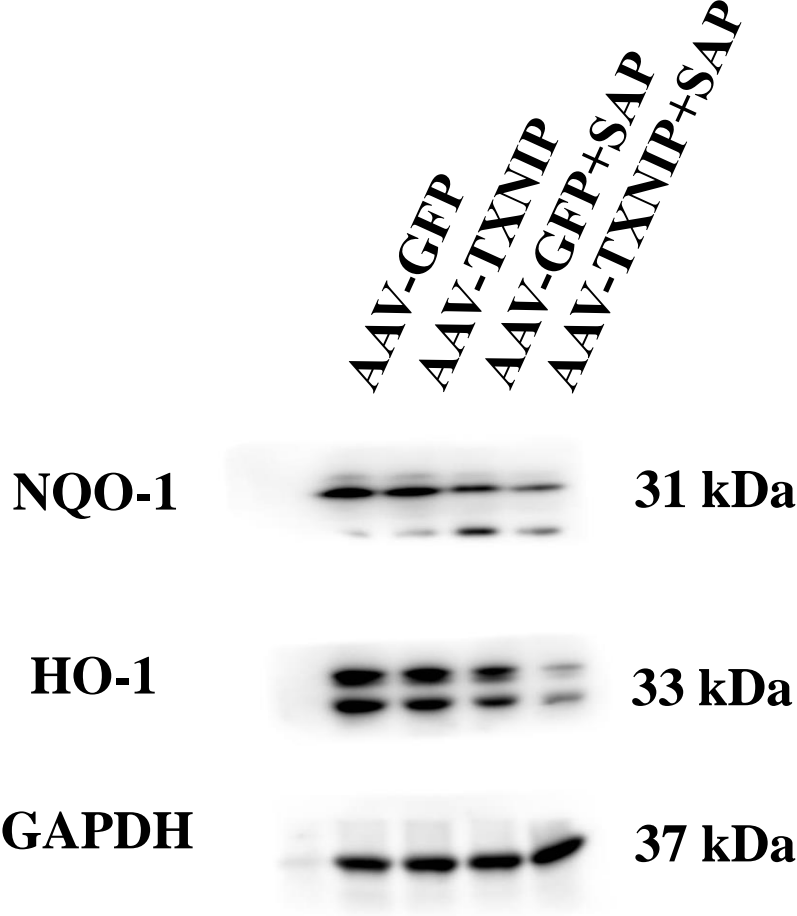

**Fig. 7.**

**A**

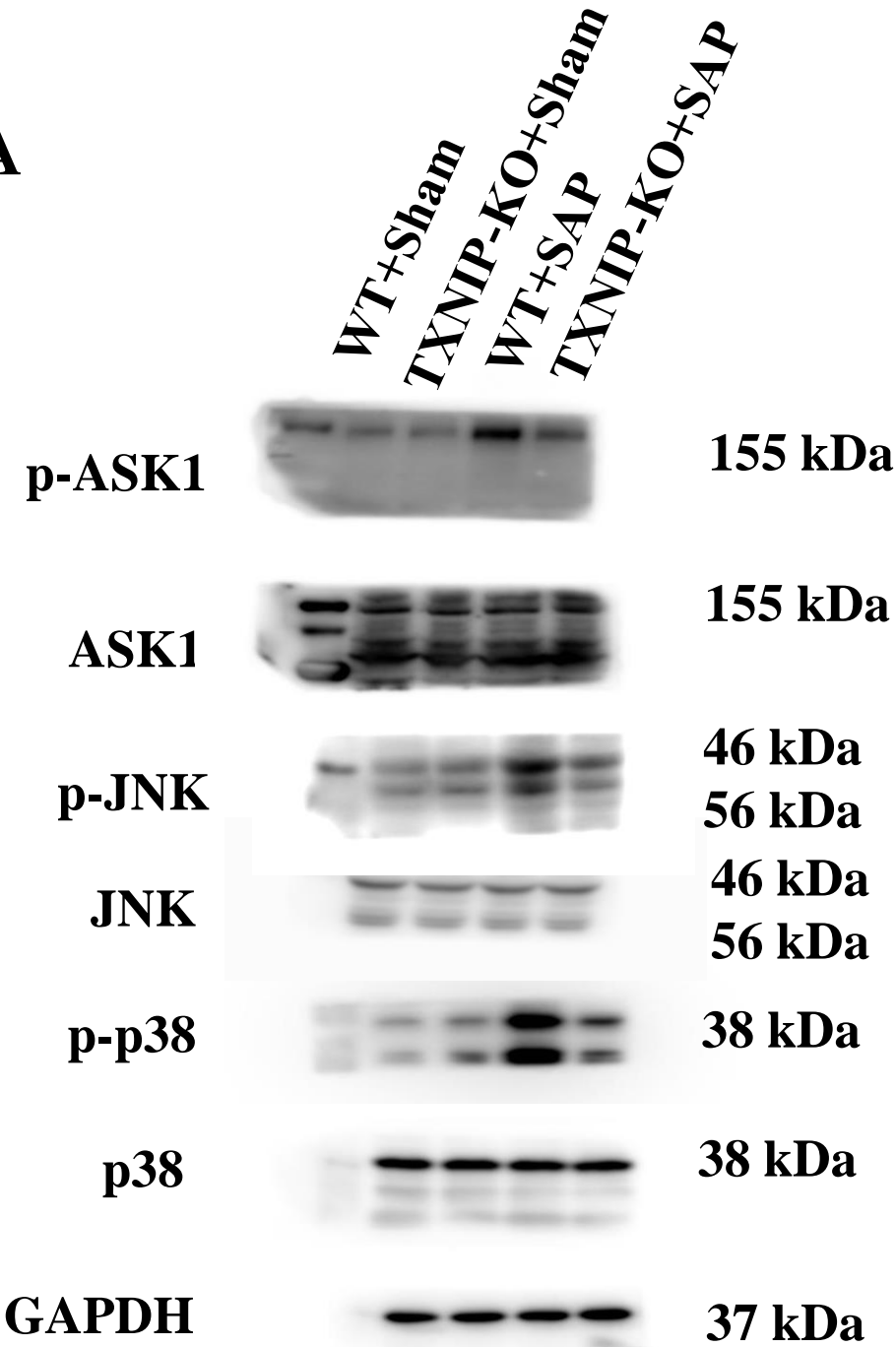

**B**

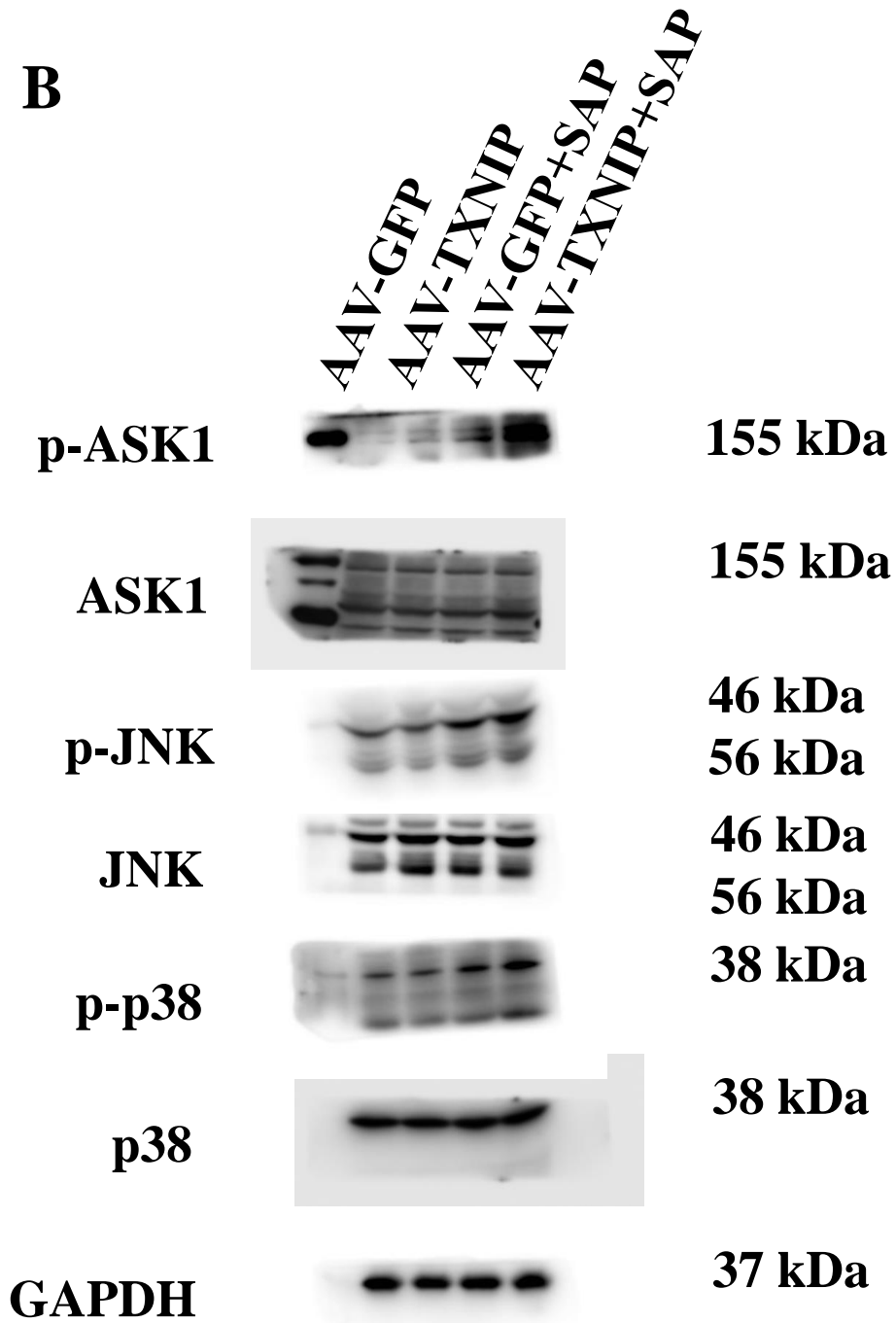

Fig. 7.

C

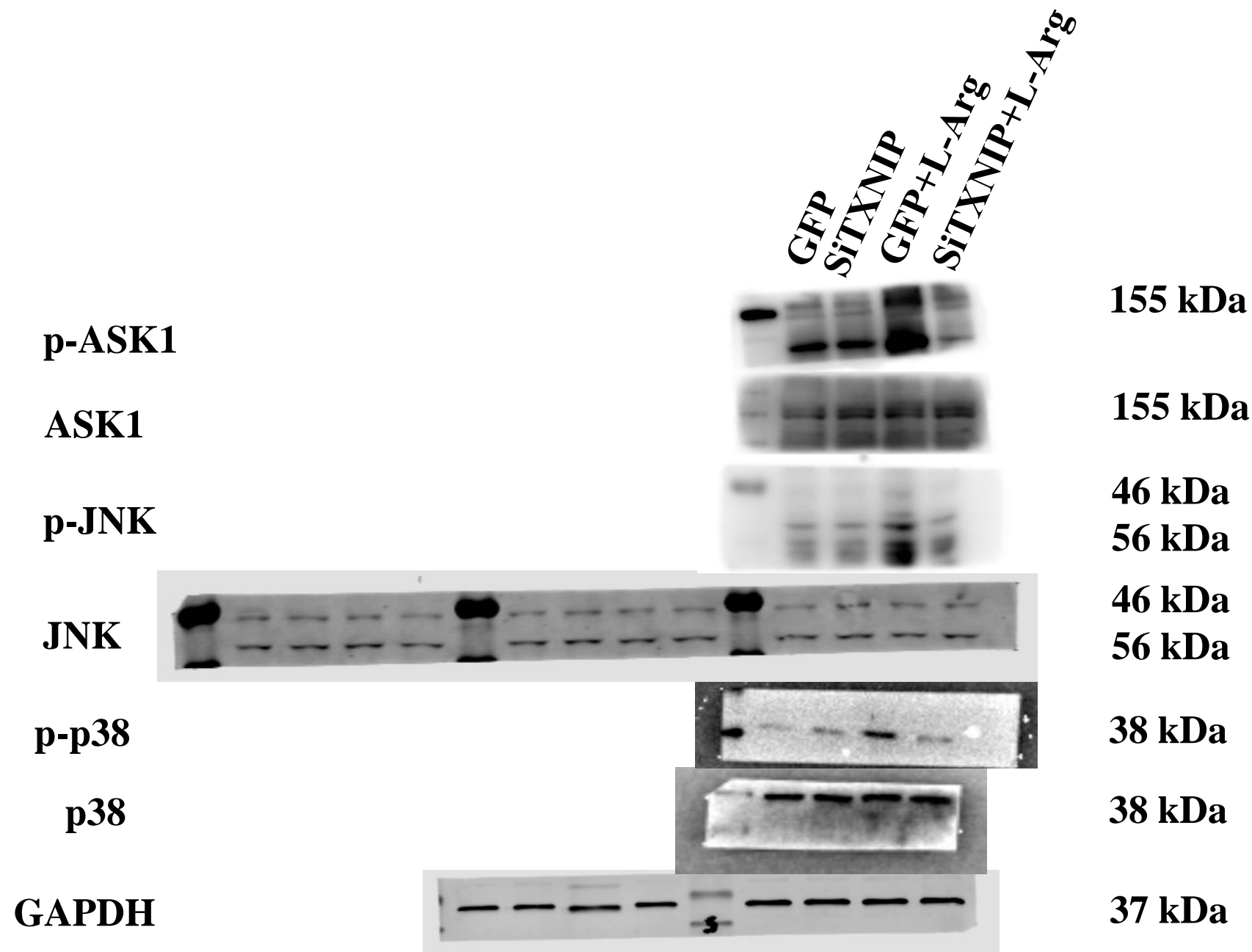

Fig. 8.

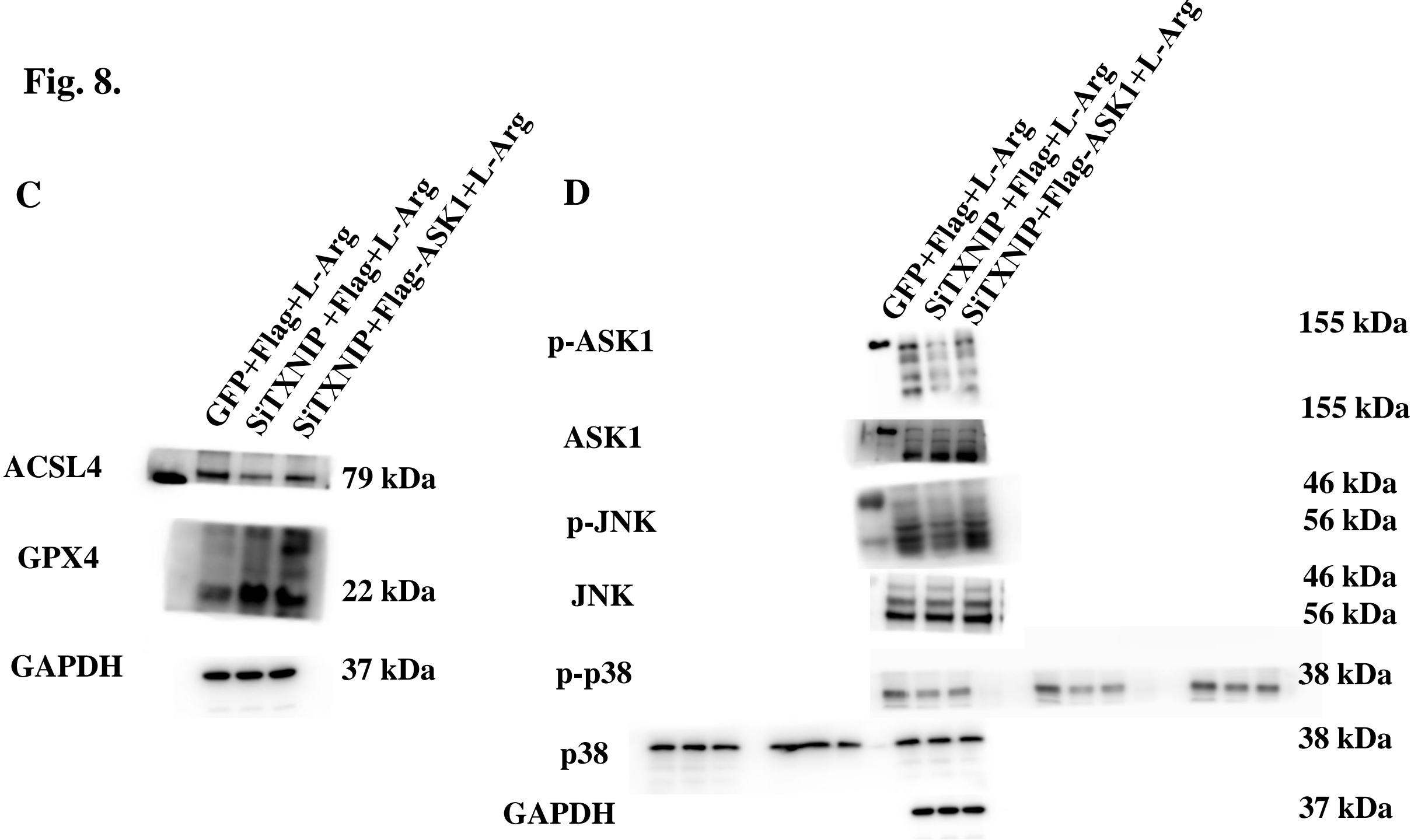

**Fig. 9.**

**F**

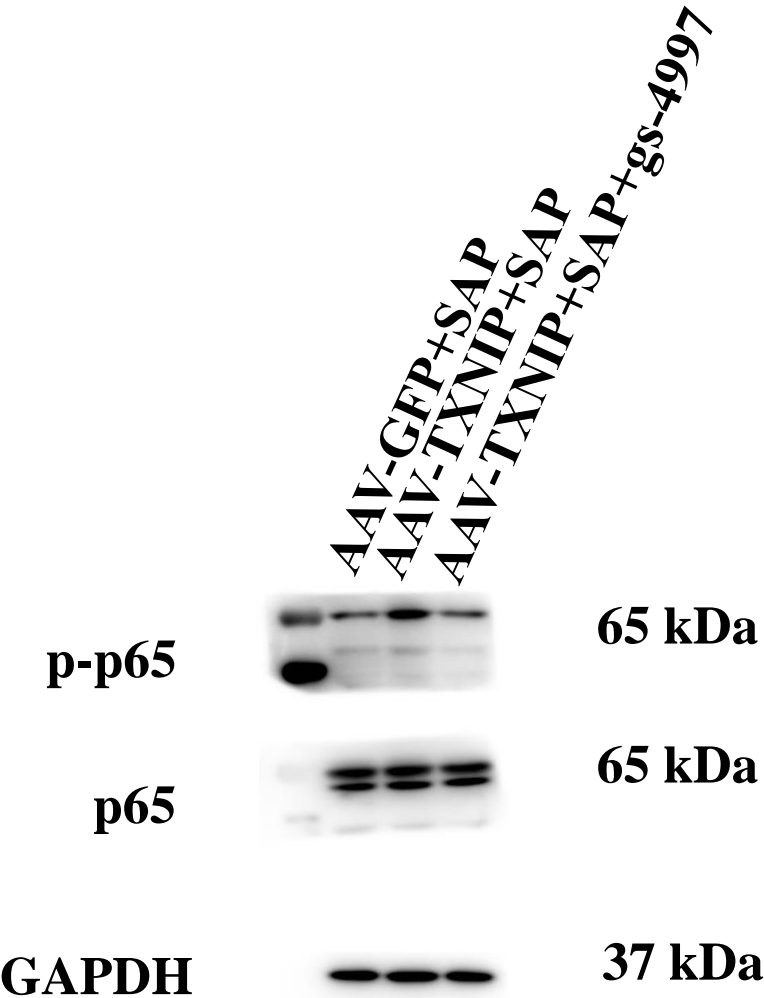

**Fig. 10.**

**E**

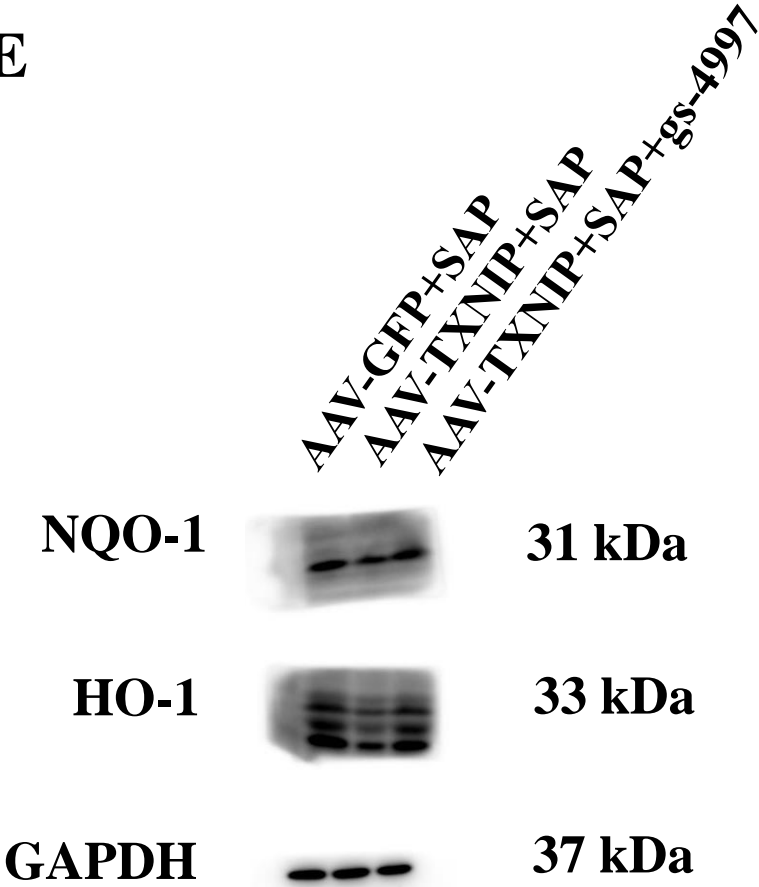

**F**

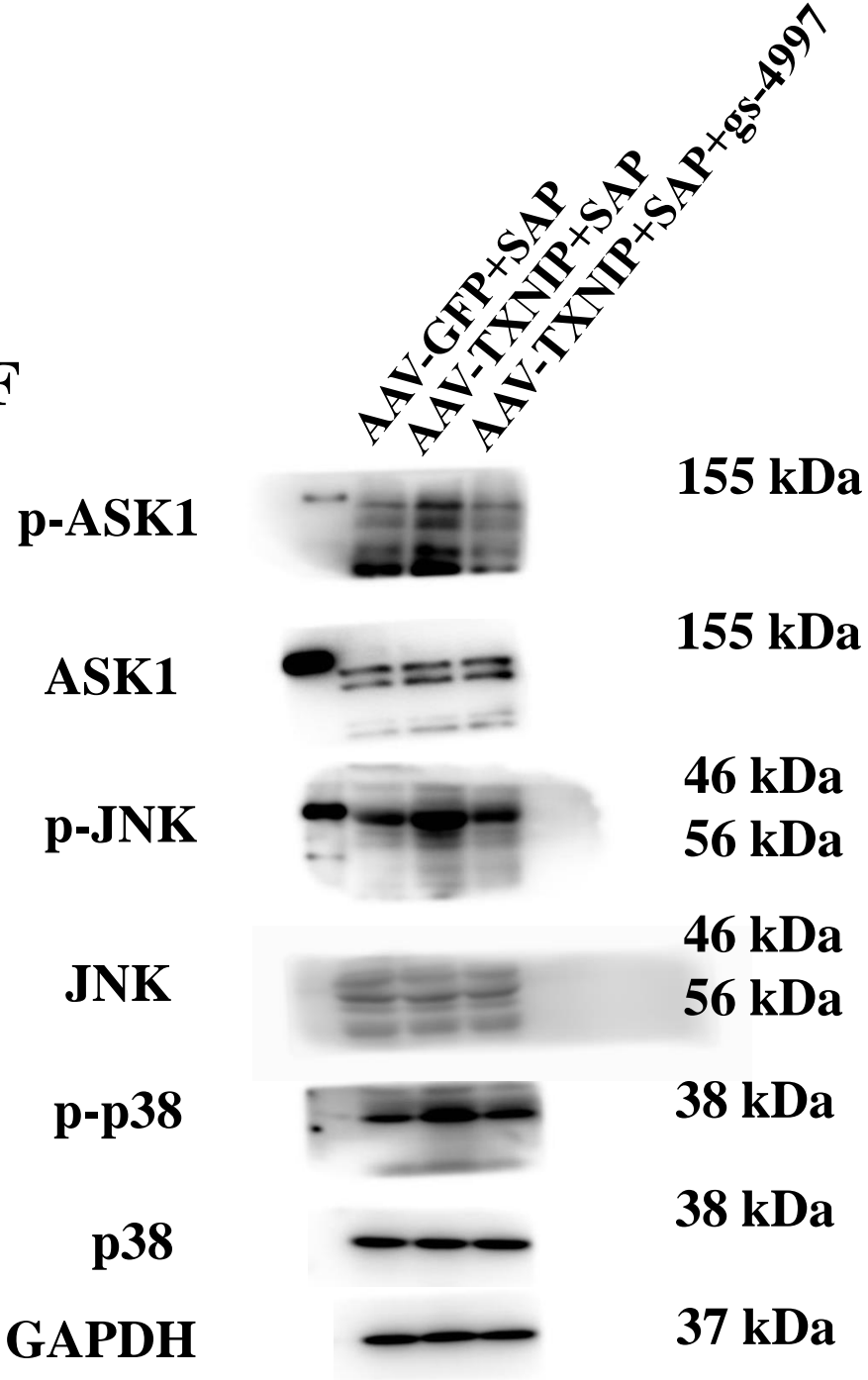

Supplement: Supplementary file 1 — Supplemental Material--Full and uncropped western blots [file 41419_2022_5355_MOESM1_ESM.pdf]
