# Supplementary material for: Thioredoxin-interacting protein deficiency protects against severe acute pancreatitis by suppressing apoptosis signal-regulating kinase 1
Source: Cell Death Dis. 2022 Oct 31;13(10):914. doi: 10.1038/s41419-022-05355-x (PMC9622726; doi:10.1038/s41419-022-05355-x)
Supplement: Supplementary file 2 — Supplementary material [file 41419_2022_5355_MOESM2_ESM.pdf]

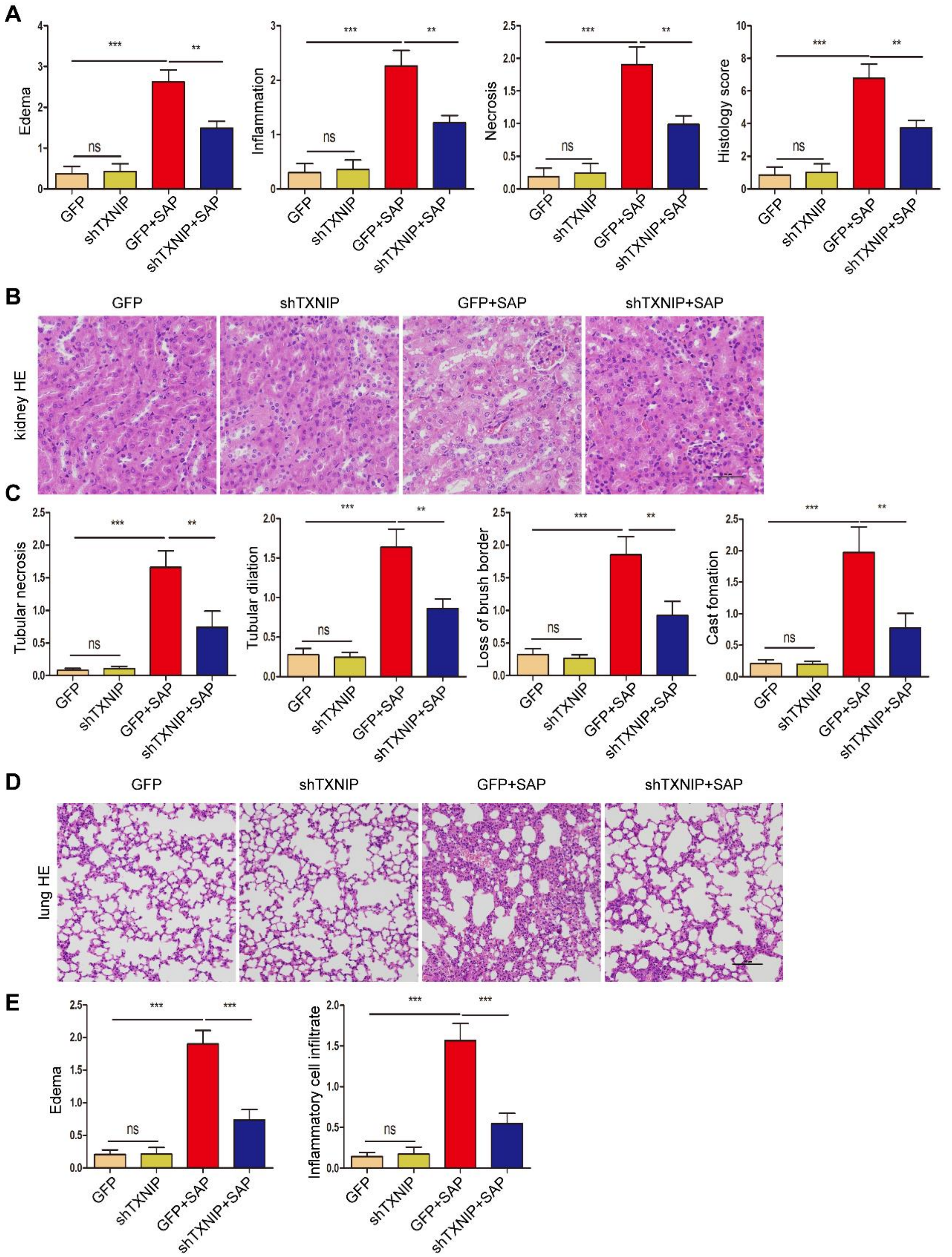


**Fig. S1. AAV-mediated TXNIP knockdown attenuated SAP in mice.** (A) Statistics showing pathological injury in the pancreas of mice in different group (n = 6 / group). (B) Representative histological H&E staining images in kidney of mice in different group. (n = 6 / group). Scale bar =50μm. (C) Statistics showing pathological injury in the kidney of mice in different group (n = 6 / group). (D) Representative histological H&E staining images in lung of mice in different group. (n = 6 / group). Scale bar

=100μm. (E) Statistics showing pathological injury in the lung of mice in different group (n = 6 / group). ** $p < 0.01$, *** $p < 0.001$, ns $p > 0.05$.

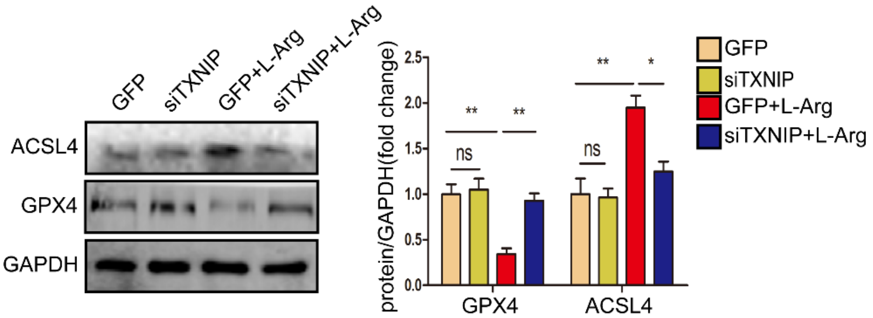


**Fig. S2. TXNIP mediates oxidative stress during SAP**. (A) Protein levels of GPX4 and ACSL4 in the AR42J cells induced by L-Arg in different group (representing three independent experiments). GAPDH as a loading control. * $p < 0.05$, ** $p < 0.01$.

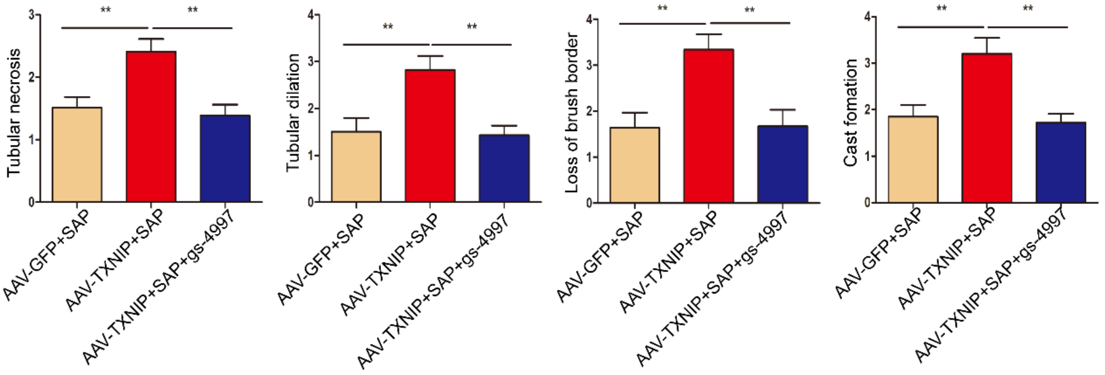


**Fig. S3. ASK1 mediated the effect of TXNIP on oxidative stress during SAP.** (A) Statistics showing pathological injury in the kidney of mice in different group (n = 6 / group). ** $p < 0.01$.
